# Detection of structural variations in densely-labelled optical DNA barcodes: A hidden Markov model approach

**DOI:** 10.1371/journal.pone.0259670

**Published:** 2021-11-05

**Authors:** Albertas Dvirnas, Callum Stewart, Vilhelm Müller, Santosh Kumar Bikkarolla, Karolin Frykholm, Linus Sandegren, Erik Kristiansson, Fredrik Westerlund, Tobias Ambjörnsson

**Affiliations:** 1 Department of Astronomy and Theoretical Physics, Lund University, Lund, Sweden; 2 Division of Chemical Biology, Department of Biology and Biological Engineering, Chalmers University of Technology, Gothenburg, Sweden; 3 Department of Medical Biochemistry and Microbiology, Uppsala University, Uppsala, Sweden; 4 Department of Mathematical Sciences, Chalmers University of Technology and the University of Gothenburg, Gothenburg, Sweden; Imperial College London, UNITED KINGDOM

## Abstract

Large-scale genomic alterations play an important role in disease, gene expression, and chromosome evolution. Optical DNA mapping (ODM), commonly categorized into sparsely-labelled ODM and densely-labelled ODM, provides sequence-specific continuous intensity profiles (DNA barcodes) along single DNA molecules and is a technique well-suited for detecting such alterations. For sparsely-labelled barcodes, the possibility to detect large genomic alterations has been investigated extensively, while densely-labelled barcodes have not received as much attention. In this work, we introduce HMMSV, a hidden Markov model (HMM) based algorithm for detecting structural variations (SVs) directly in densely-labelled barcodes without access to sequence information. We evaluate our approach using simulated data-sets with 5 different types of SVs, and combinations thereof, and demonstrate that the method reaches a true positive rate greater than 80% for randomly generated barcodes with single variations of size 25 kilobases (kb). Increasing the length of the SV further leads to larger true positive rates. For a real data-set with experimental barcodes on bacterial plasmids, we successfully detect matching barcode pairs and SVs without any particular assumption of the types of SVs present. Instead, our method effectively goes through all possible combinations of SVs. Since ODM works on length scales typically not reachable with other techniques, our methodology is a promising tool for identifying arbitrary combinations of genomic alterations.

## Introduction

Optical DNA mapping (ODM) provides a sequence-specific fluorescence “fingerprint” (DNA barcode) for single DNA molecules, which is well suited for analyzing ultra-long DNA molecules (> 10^5^ basepairs (bp) long). The barcodes are created by fluorescent labelling of individual DNA molecules in a sequence-specific manner, stretching the molecules using nanochannels or surface adsorption, and imaging them using a fluorescence microscope [1]. Currently, the most common approach of DNA labelling is sparse enzymatic labelling. The output of this approach is an array of sequence-specific “dots” along the DNA. An alternative approach is dense labelling ODM, with examples including dense enzymatic labelling with methyltransferases [2], DNA melt mapping [3], and competitive binding (CB) [4]. When using dense labelling, individual dots are not discernible (the resolution of a single dot is described by a point spread function with a width *σ*_*psf*_, typically around 1 kb) and, rather, the output is a sequence-specific continuous intensity profile (barcode) along the DNA.

Note that DNA barcodes can be predicted using DNA sequences as input. However, DNA barcodes can also be used as stand-alone sequence-specific “fingerprints” which do not rely on DNA sequence information. In this study, we are investigating the latter case, i.e., the use of DNA barcodes as stand-alone fingerprints.

A DNA barcode contains information of larger genomic alterations along the DNA, which are referred to as structural variations (SVs) [5]. There are several types of SVs, including insertions, deletions, inversions, repetitions, and translocations, and each of these are visible directly in the results from the densely-labelled barcodes of long DNA molecules without access to sequence information.

SV-detection using sparsely-labelled barcodes has been investigated extensively previously [6–9], and new methods are being continuously developed to make the detection more efficient and compatible with various experimental techniques [10]. The data produced by densely-labelled ODM has, however, not received as much attention with respect to SV-detection. In this work, we provide tools for SV-detection in densely-labelled barcode analysis that complements those which already exist for sparsely-labelled barcodes.

In this study, the application of our new SV method involves plasmids, mobile genetic elements that enable the spread of antibiotic resistance genes between bacteria. Antibiotic resistance genes encoded on plasmids are often flanked by mobile genetic elements (insertion sequences, integrons, transposons) and can have a high rate of transfer creating frequent insertions and rearrangements as well as exchange of DNA between different plasmids or between plasmids and chromosomes [11]. This makes SVs very common on plasmids and analysis of these are important for identification of novel combinations of resistance genes and understanding the evolution of resistance plasmids. Rapid identification of how and when plasmid transfers occur is also of importance in forming efficient countermeasures preventing the spread of antibiotic resistance.

The CB DNA labelling method, from which all experimental data in this study originate, is an enzyme-free densely-labelled ODM assay based on the competitive binding of two small molecules, YOYO-1 (fluorescent) and netropsin (non-fluorescent), to DNA molecules [12]. This method has been extensively used for plasmid analysis, both to identify plasmids from sequence databases [13] and to investigate possible spread of resistance in hospitals [14–16]. Even if our experimental data is from the CB assay, we point out that our methodology is suitable for the other types of densely-labelled barcodes as well.

Most previous ODM-based methods for comparing densely-labelled barcodes compared intact barcodes assumed to have no SVs or have just single insertions or deletions. To compare densely-labelled barcodes without any SVs to each other or to a database of theoretical barcodes (calculated using previously sequenced DNA as input), it is then sufficient to use a correlation-based approach [5]. However, to compare barcodes with complex combinations of SVs and without having a database as a reference is challenging, as neither the types nor the lengths of the SVs are known.

A few previous methods for comparing densely-labelled barcodes with SVs exist. A study using melt mapping (DNA denaturation) introduced a sliding-window analysis method [17, 18]. This method compares pieces (barcodes) of the molecule’s experimental denaturation map to in silico maps computed from the reference genome to detect SVs. Using the sliding-window analysis method, insertions and deletions down to 5 kb could be detected with a high confidence. However, the approach uses a theoretical barcode computed using a reference genome, can not detect inversions or translocations with the same confidence as insertions and deletions, and the existence of SVs has to be validated visually. Therefore, remaining challenges for detecting SVs include making the analysis automated, implementing it for more general SVs, and dealing with the case when no reference DNA sequence is available.

In order to address the remaining limitations of previous densely-labelled-ODM methods for comparing barcodes with SVs, we here introduce a Hidden Markov Model (HMM) based approach to solve the problem of detecting SVs in DNA barcodes. This approach borrows ideas from “Multi Segment Viterbi”-based bio-informatics tools for protein alignment [19] in order to compare a query barcode to a reference barcode. In this comparison we do not differentiate between experimental and theoretical reference barcodes, therefore we overcome the limitation of requiring a reference genome DNA sequence. We interpret the alignment of the two barcodes as an optimal path through the hidden states of an HMM. The space of all paths corresponds to all the possible alignments between the two barcodes. Based on our findings in a previous study of contig assembly using ODM, we require that each of the aligned sub-barcode pairs of the two barcodes has to be longer than approximately 22 kb [20]. This length constraint is incorporated directly into our HMM, thereby extending the approach in [19] where no such constraint is used. The output of our HMM is then post-processed in an automated way with the help of a matrix profile [21] and p-value threshold in order to overcome the need for visual inspection of the results. Our method can detect all 5 types of SVs, and any combination thereof, provided that the path through corresponding states is significant. In this way we address the previously described limitations of other approaches.

We foresee that a potential future use of our HMM pipeline is to match barcodes of non-sequenced plasmids to a database of theoretical barcodes. For regions that match we can then obtain the sequence of parts of the non-sequenced plasmid by directly retrieving it from the match in the database.

## Materials and methods

**Fig 1:**
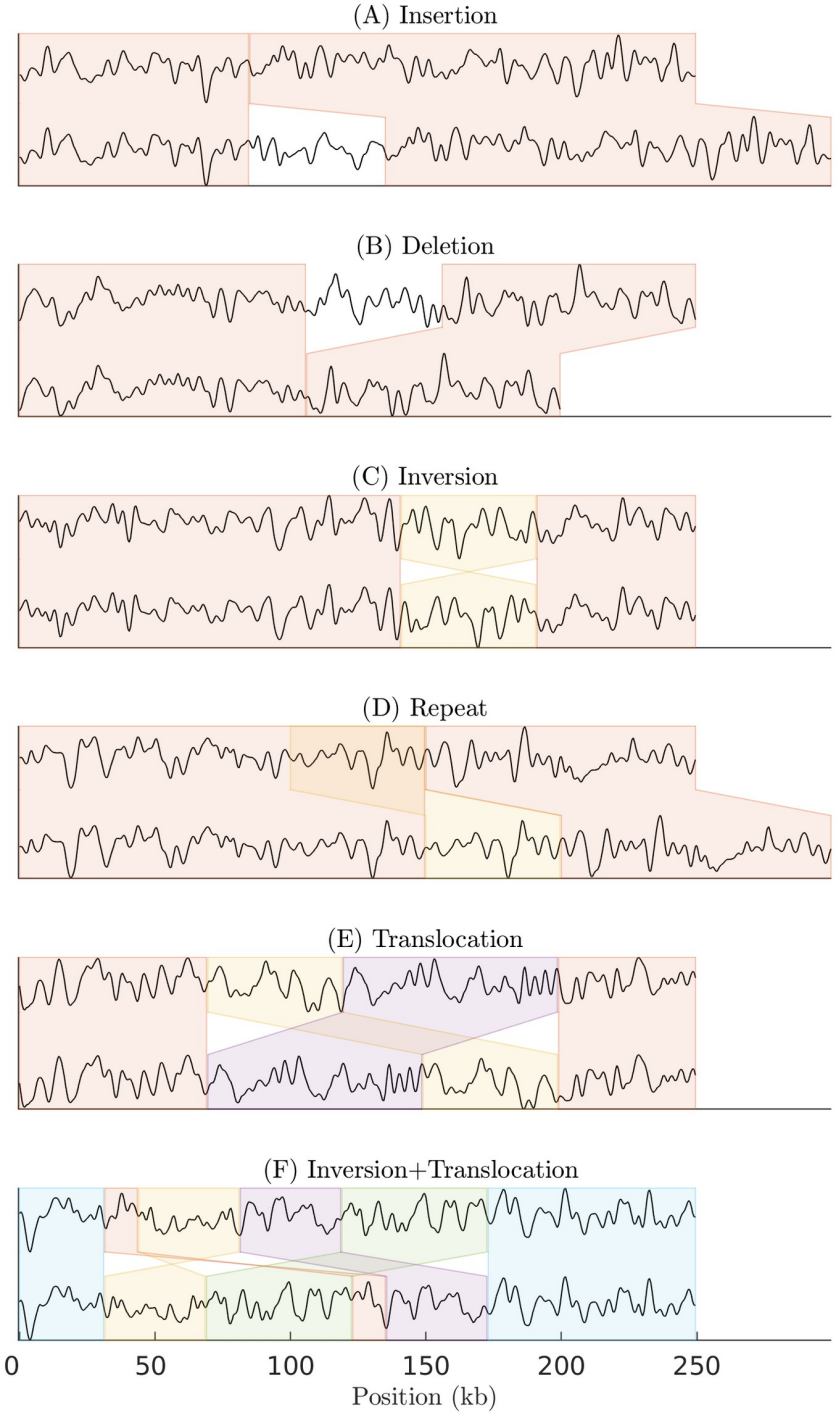
Schematics of the structural variations (SVs) problem using DNA barcodes. As an illustration of different types of SVs, shown here are 6 different pairs (above: reference barcode, below: query barcode) of stacked barcodes: (A) An insertion, a sub-barcode is inserted in the query barcode. (B) A deletion, a sub-barcode is deleted in the query barcode. (C) An inversion, involves flipping a sub-barcode in the query barcode. (D) A repeat, a sub-barcode is repeated two (or more) times. (E) A translocation, a sub-barcode in the query barcode is moved to a different place on the reference barcode. (F) Inversion+Translocation, a complex SV involving both flipping a sub-barcode in the query barcode and moving a sub-barcode in the query barcode to a different place compared to the reference barcode. In these examples all query barcodes are random barcodes (see Table 1) of 500 pixels (≈250 kb) length and the SVs are 100 pixels (≈50 kb) long. Matching sub-barcodes are enveloped in boxes of the same colour.

**Fig 2:**
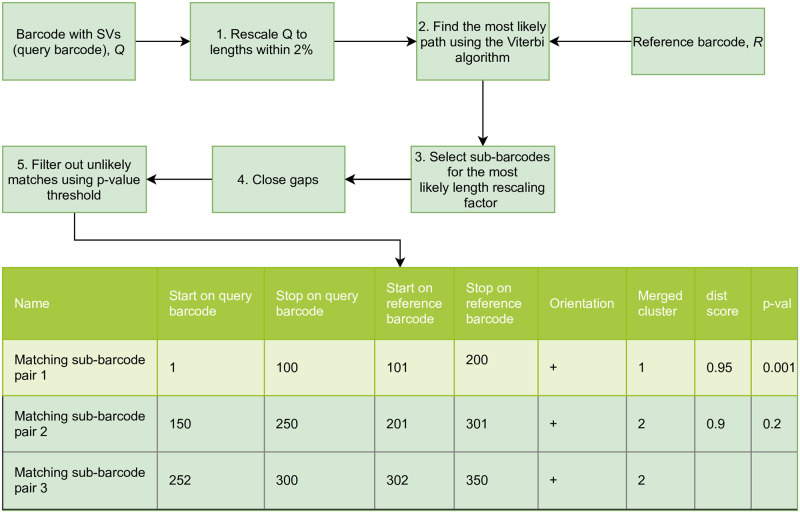
Hidden Markov Model (HMM) approach for detecting SVs in barcodes. The method consists of 5 steps: 1) The length of the query barcode (barcode with SVs) is rescaled based on a range of length re-scaling factors around an initial estimate of length re-scaling factor. 2) The most likely path through the states, which defines the final alignment, is found using Viterbi algorithm. This path corresponds to pairs of indices of sub-barcodes between query and reference barcodes. 3) Sub-barcodes based on the most likely length re-scaling factor are selected. 4) Gaps and overlaps that are separated by a distance no more than *g* are closed (sub-barcodes merged). 5) Unlikely matches are filtered out using a p-value threshold *p*_thresh_. Finally, the output table with the detected matching sub-barcode pairs is given.

We consider the problem of identifying structural similarities and differences between two DNA barcodes (See Fig 1). The first of these barcodes is used as a query and is called the query barcode, while the second barcode is called the reference barcode. These barcodes may have one or more SVs (insertion, deletion, inversion, translocation or repetition). We developed a method (See Fig 2) to identify these structural differences. The output of our method is a set of pairs of sub-barcodes. A sub-barcode pair is a local alignment of a sub-barcode of the query barcode to a sub-barcode on the reference barcode. Definitions of barcodes, sub-barcode and other relevant terms are found in Sec. 1 in S1 Text.

### Datasets

In the first part of this study (see “Results”) we use “noisified random SV barcodes”, see Table 1. These barcodes are, for practical purposes, similar to experimental barcodes, but have the added value that we know exactly where the SVs are (the “ground truth” is known). To generate noisified random SV barcodes, we first generate random barcodes by convolving an array of random numbers with the optical point spread function (PSF) of the system. We then noisify the random barcodes in order to mimic the effect of shot noise and other types of experimental sources of errors. We finally add one or more SV to the noisified random barcodes, thereby generating noisified random SV barcodes. The location of the added SV is noted in a table so that it could be later used to calculate which parts of the barcodes were matched correctly using the our methods. Further details are found in the Secs. 3.1 and 3.2 in S1 Text.

**Table 1:** List of the different types of DNA barcodes used in this study.

Name	Explanation/Source
Individual experimental barcode	Time-averaged intensity profile
	from a single DNA molecule
Experimental consensus barcode	Average of several individual experimental
	barcodes / Table 1 in S1 Text
Theoretical barcode	ODM barcode calculated from microscopic theory,
	using a DNA sequence as input,
	then convolved with a PSF
Random barcode	Array of Gaussian random numbers
	convolved with a PSF
Synthetic barcode	Theoretical barcode + noise
SV barcode	Theoretical barcode + structural variation (SV)
Synthetic SV barcode	Synthetic barcode + SV
Noisified barcode	Random barcode + noise
Random SV barcode	Random barcode + SV
Noisified random SV barcode	Random SV barcode + noise

The procedure for generating experimental barcodes is detailed in [13]. A theoretical competitive binding barcode is calculated using the transfer matrix method from [20]. The PSF is a Gaussian of width *σ*_*psf*_. Noise adds local fluctuations around the intensity values of the barcode, controlled by the parameter *noiseLevel* (which is equal to 1 − *dist* value between noisified barcode and barcode without noise) and described in Sec. 2.1 in S1 Text. The five SV types—insertion, deletion, inversion, repetition or translocation are described in Sec. 2.2 in S1 Text.

In the second part of the study (see “Results”) we compare experimental consensus barcodes against other experimental barcodes and theoretical barcodes. The details on how to generate experimental barcodes are found in [13]. The details on how theoretical barcodes are obtained, using a DNA sequence as input, are described in [20].

### Analysis pipeline

We applied a Hidden Markov Model (HMM) based approach [22] for detecting SVs (see Fig 1) in densely-labelled DNA barcodes. In short, two barcodes of lengths *q* and *d* are aligned to each other using a Viterbi algorithm for a probabilistic length-constrained HMM [23] containing 2*q* + 2 states. The method has hyper-parameters *p*_*MM*_, *p*_*GG*_ (representing the probabilities for jumping between different states, where *M* represents a match state and *G* represents gap state), *l* (minimum match length constraint) and *l*_*G*_ (minimum gap length constraint).

Our method involves five steps as schematically illustrated in Fig 2 and described in more detail below:

(1) In the first step a real valued query barcode *Q* = {*a*_1_, …, *a*_*q*_} is length re-scaled (the reference barcode *D* = {*b*_1_, …, *b*_*d*_} is fixed), because although barcodes are averages of repeated fluorescence measurements of the same type of DNA molecule, separate DNA molecules (even of the same type) are extended to slightly different lengths in ODM experiments. To deal with this, we consider different length re-scaling factors (within 2%) for barcodes around the initially estimated length re-scaling factor. In the case where the initial length re-scaling factor is not known, we compare length re-scaled barcodes *Q* against *D* using a matrix profile algorithm called STOMP [24] to get the length re-scaling factor estimate (See Sec 3.7 in S1 Text).
(2) Next, we find the most likely path between the reference barcode *D* and the query barcode *Q* using a constrained Viterbi algorithm. We do not want to consider all possible paths, since we do not want to match small (less than 22 kb long [20]) sub-barcodes. We therefore use constraints for the minimum number of consecutive match states *l* and for the minimum number of consecutive gap states *l*_*G*_ ($l,l_{G}\in N_{\geq 0}$). Consecutive elements in the most likely path maps sub-barcodes of the query barcode *Q* to sub-barcodes of reference barcode *D*.
(3) (After repeating (2) for all length re-scaled query barcodes, we choose the SV result for the length re-scaling factor which had the maximum value of a similarity measure, *dist* (in this study we use Pearson Cross Correlation as the similarity measure) for the longest extracted sub-barcode weighted by its length.
(4) Using the result from step (3), we then close gaps in the alignment. To that end, we merge sub-barcodes which are found by the HMM if their edges are within *g* pixels on both the query and the reference barcodes. Since the resolution of the barcode is set by *σ*_*psf*_ (see Introduction) gaps of this size are not “physical”; the Viterbi algorithm does not involve the correlations over a length of the order *σ*_*psf*_, so this is a way of compensating for this effect.
(5) Finally, we set a p-value threshold *p*_thresh_ = 0.01 for describing which detected merged sub-barcode pairs are significant. For each pair of merged sub-barcodes of length *L* detected by the HMM alignment, a *dist* score is computed. This score is converted to a p-value based on the distribution of dist-scores for random barcodes of the same length. The p-value is then compared to *p*_thresh_ and the sub-barcode pair is discarded if the threshold is not passed.

As an output of alignment of the query barcode to the reference barcode, we get pairs of matching merged sub-barcodes, which are then output in an alignment table, together with a *dist* score for each pair of merged sub-barcode pair, see bottom of Fig 2 for an example. The details of each of the 5 steps in our method, and parameter values, are found in the Sec. 3 in S1 Text

Our software is publicly available as a MATLAB package “hmmsv”, see Data availability statement at the end of this article.

### True positive rate

The true positive rate (TPR) is a rate that an actual pixel match between the query and reference barcodes will show up as a match also in our HMM output. When estimating the true positive rate for the HMM output of a comparison of the query barcode *Q* against the reference barcode *D* in the case of random SV barcodes, we use the known ground truth alignment table (which contains the alignment of matching sub-barcode pairs). Given the HMM output, for each pixel of the query (1, 2, …, *q*), we create a binary matrix which has a non-zero value in *i*th row and *j*th column only if pixel *i* from the query is matched to pixel *j* from the reference in the HMM alignment table. Formally, the elements of the binary matrix are:

$$\begin{array}{c} m_{i,j}=\{\begin{array}{c} 1\;\text{if}\;\left[i,j\right]\in \left\{p_{1},p_{2}\right\}\\ 0\;\text{otherwise} \end{array},\;i=1,2\ldots ,d,j=1,2,\ldots ,q, \end{array}$$ (1)

where {*p*_1_, *p*_2_} represents the set of all pixel pairs matched in the output of HMM procedure and *d* is the length of reference, as before. Similarly, the ground truth alignment table is represented by a binary matrix with elements

$$\begin{array}{c} t_{i,j}=\{\begin{array}{c} 1\;\text{if}\;\left[i,j\right]\in \left\{t_{1},t_{2}\right\}\\ 0\;\text{otherwise} \end{array},\;i=1,2\ldots ,d,j=1,2,\ldots ,q, \end{array}$$ (2)

where {*t*_1_, *t*_2_} represents the set of all pixel pairs in the ground truth alignment table.

Once the two match tables above have been generated, we iterate through the set of non-zero elements in each row of the ground truth matrix *t*_*i*,*j*_. When considering the element in column *k* in row *i*, i.e. *t*_*i*,*k*_, if there is a non-zero element *m*_*i*,*u*_ with *u* = *k* −1, *k*, *k* + 1, we consider that (*i*, *k*) is a true pixel match. We then set *m*_*i*,*u*_ = 0 (so that we would not match the same pixel twice), and continue iterating through the non-zero elements of the alignment table. From this iterative procedure, we obtain the number of true positives (TP), i.e., the number of true pixel pair matches, and the number of false negatives (FN), i.e., the number of pixels pairs that are not considered true pixel matches. The true positive rate is then estimated as

$$\begin{array}{c} truePositiveRate=\frac{TP}{FN+TP}. \end{array}$$

In the Sec. 2.9 in S1 Text, we also describe how to calculate false positive rates (FPR). In brief, we use random barcodes, see Table 1, to compute the number of false positives and true negatives. The FPR is used, together with the TPR, when we tune the parameters in the HMM method.

## Results

In this section we apply our HMM-based SV-detection pipeline to noisified random SV barcodes and experimental barcodes to find significantly similar sub-barcodes. Realistically looking noisified random SV barcodes were generated as described in the Methods section.

We first determined the parameters for the HMM model. To that end, we ran our HMM model with gridded parameter values for noisified random SV barcodes in order to generate true positive and true negative rates. Averaging the rates for 100 barcode pairs, we create heat-maps of true positive and true negative rates. TPR and FPR heat maps before and after p-value thresholding are found in S5 and S6 Figs in S1 Text. We use these to make a final choice for the constants *p*_*MM*_ and *p*_*GG*_. A good choice for parameters *p*_*MM*_ and *p*_*GG*_ would be where we maximize the true positive rate, while keeping the true negative rate non-zero. However, since our method is complemented by p-value thresholding, most false positives are discarded using post-processing, and therefore we make the parameter selection based on the true positive rate. We selected *p*_*MM*_ = 0.51 and *p*_*GG*_ = 0.31. The details are found in Sec. 3.11 in S1 Text. The full list of parameters is found in Table 4 in S1 Text.

**Fig 3:**
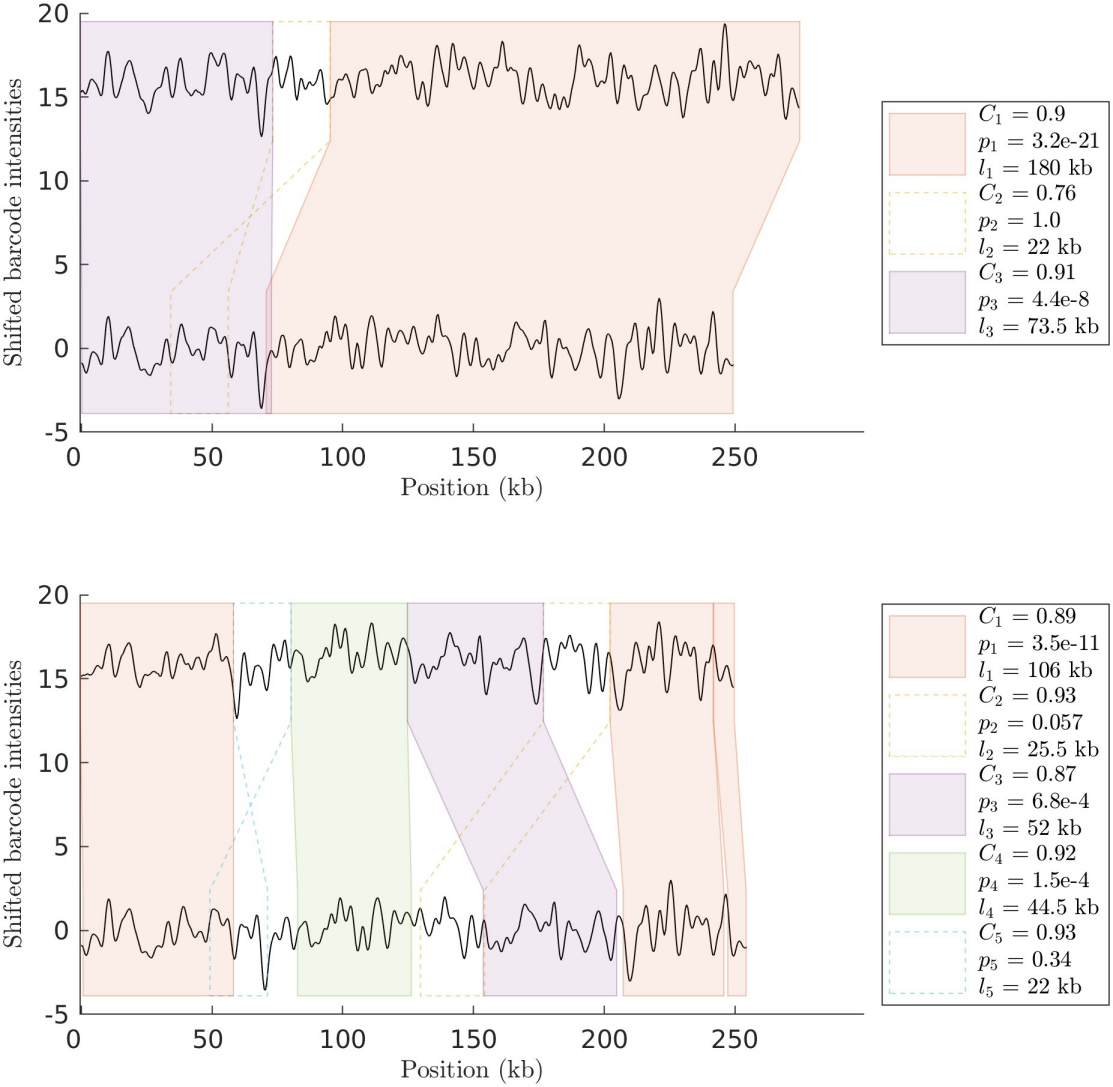
SV-detection for noisified random SV barcodes. (Top) HMM output for comparison of two noisified random SV barcodes with a single 50 pixel (25 kb) insertion. (Bottom) HMM output for comparison of two noisified random SV barcodes with a 50 pixel (25 kb) inversion and a 50 pixel (25 kb) translocation. Sub-barcode pairs that did not pass the p-value threshold are visualized in dashed boxes. In the tables next to each figure, *dist* scores for sub-barcodes *C*_*i*_, p-values *p*_*i*_, and sub-barcode lengths *l*_*i*_ are reported. The noise level, 1 − *dist*, was here set to 0.1.

Next we make a comparison of the output of the HMM method for noisified random SV barcodes (as defined in Table 1). In Fig 3 (top) we show a barcode with a single insertion of size 25 kb matched against the query barcode. Note that our analysis pipeline correctly identifies the insertion (for the noise level used). Fig 3 (bottom) shows an example with a more complicated type of SV. Here, a 250 kb random query barcode was matched against a noisified random SV barcode containing both an inversion and a translocation. Again, our analysis pipeline gives a correct output when compared to the ground truth.

**Fig 4:**
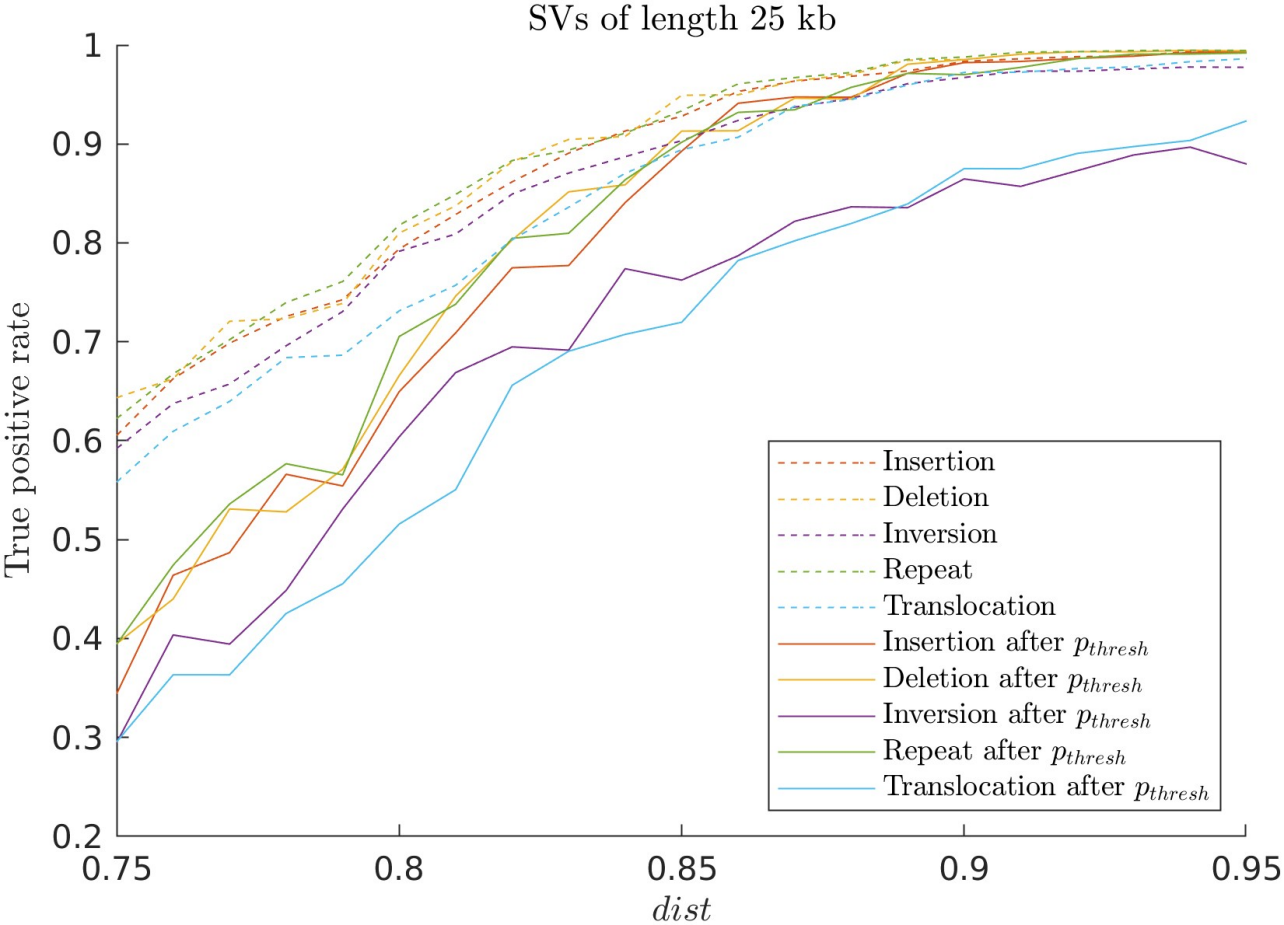
Dependence of true positive rate on noise in noisified random SV barcodes of different SVs. We evaluate the five different SVs (insertion, deletion, inversion, repeat, and translocation) with random query and reference barcodes to test how true positive rate depends on the presence of different levels of noise. The associated figure showing the TPR as a function of the lengths of the SVs is found in S7 Fig in S1 Text. We find that the success rate (here measured by a true positive rate) is close to 0 after the p-value threshold for smaller values of *dist* (the noise is quantified by the *dist* value between noisified random SV barcode and random SV bacode without noise), but gets closer to 1 for larger values of *dist*. We used 100 pairs of random query (250 kb) and noisified random SV data barcodes with SVs of length 25 kb for *dist* ranging from 0.75 to 0.95.

We then investigated how the accuracy of the method relates to the amount of noise present in the query and reference barcodes. Noise was added to the reference barcode as described in Table 1, and quantified by the noise level, 1 − *dist*. We evaluated the accuracy of the model by calculating a true positive rate before and after applying a p-value threshold to the output of the noisified random SV barcodes comparison. We used five different types of SVs with a single SV (Fig 4). We found that the performance rate (here measured by a true positive rate) was close to 0 after the p-value threshold for small values of *dist*, but got closer to 1 as we increased *dist* (decreased the noise levels). In the best case, *truePositiveRate* = 1, meaning that there were no false negatives, but it will not be 1 as soon as there are random components. Typical values for the *dist* score when comparing plasmids of length 200–250 kb to theory was *dist* = 0.9 − 0.95 [20]. For noisified random SV barcodes having such *dist* scores, the *truePositiveRate* > 0.85 even after removal of some of the sub-barcodes by the p-value threshold.

**Fig 5:**
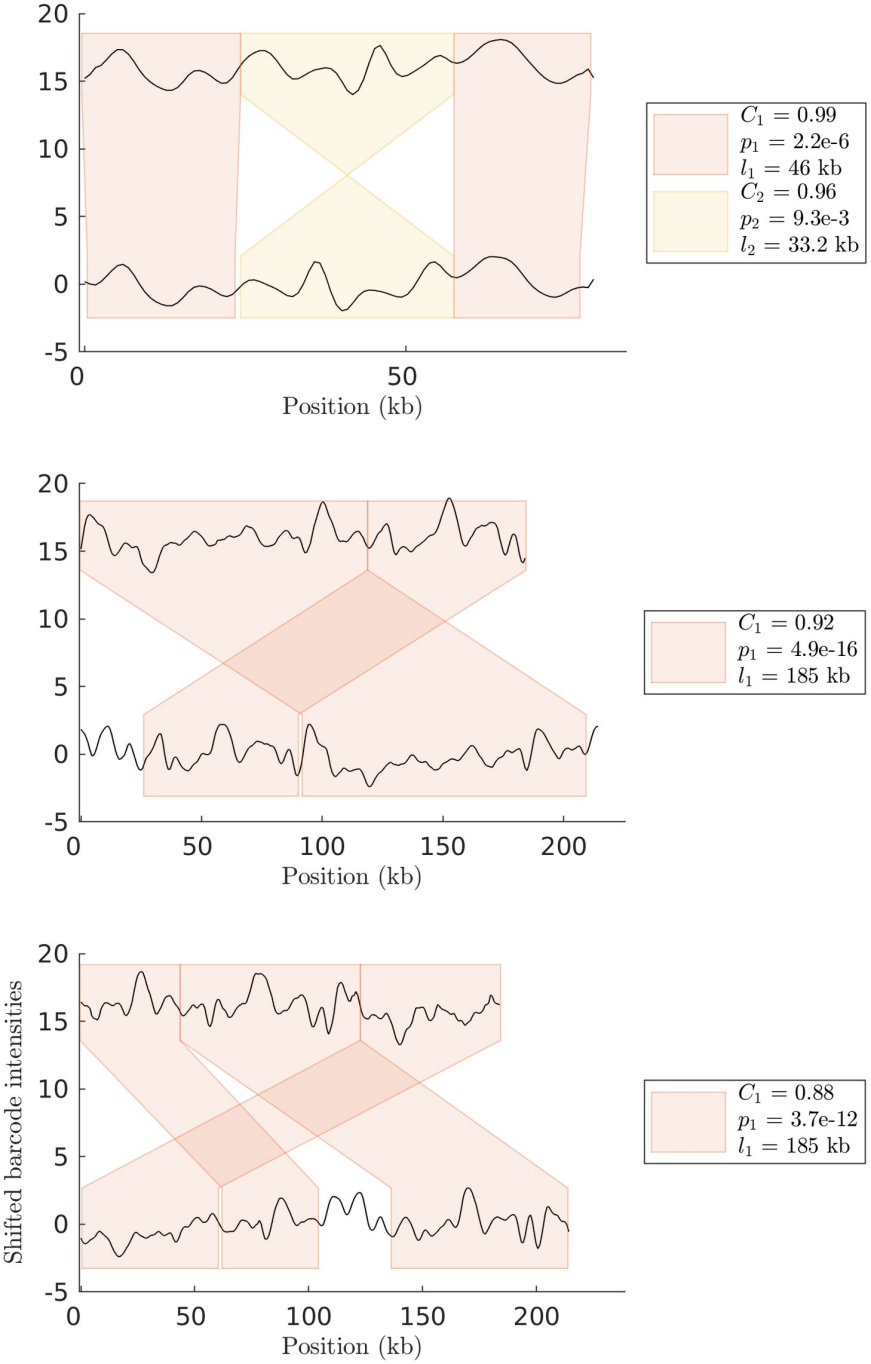
HMM output for real data from a neonatal outbreak. (Top) Output of the HMM method for comparison of two experimental ESBL-KP 80 kb consensus barcodes. Detected sub-barcode pairs suggest that there was a roughly 33 kb inversion in the middle. (Middle) Output of the HMM method for comparison of two experimental 215 kb consensus barcodes from different patients taken at approximately the same time. We find that all smaller sub-barcodes have been merged together, and there is a deletion (30 kb) on the reference barcode. (Bottom) Output of the HMM method for comparison of two experimental 215 kb consensus barcodes which shows a change that occurred within a patient over a 2 years period. Same color boxes contain significantly matching sub-barcodes. The detected sub-barcode has a *dist* score *C*_*i*_, p-value *p*_*i*_, and is of length *l*_*i*_.

We next demonstrate the usefulness of the of the HMM pipeline to automatically, and without assuming a particular SV type, detect SVs for two clinically important scenarios using experimental data on bacterial plasmids. We recently demonstrated how ODM can be used to trace bacterial plasmids in an outbreak of multi-resistant bacteria at two neonatal wards at Karolinska university hospital [15]. 16 neonates were colonized by multi-resistant *Klebsiella pneumoniae* bacteria and using ODM we demonstrated that all of them carried the same two plasmids (80 kb and 215 kb) and that the plasmids remained similar for up to two years. By visual inspection of the ODM data we could observe several SVs in the plasmids. One example is the smaller plasmid in two samples collected from the same patient 25 months apart. Visual inspection suggests that a large inversion has occurred and indeed the HMM pipeline automatically identifies this inversion and shows that it is 33 kb in size (See Fig 5 (Top)). If the inversion is smaller, it is still possible to detect that there is an SV, but not to automatically identify it as significant, as seen in another example in S9 Fig in S1 Text. For the larger plasmid we found deletions in several cases and we successfully identified deletions of sizes 30 kb (See Fig 5 (Middle, Bottom)), 5 kb (S8 Fig in S1 Text (Top)) and 68 kb (S8 Fig in S1 Text (Bottom)).

**Fig 6:**
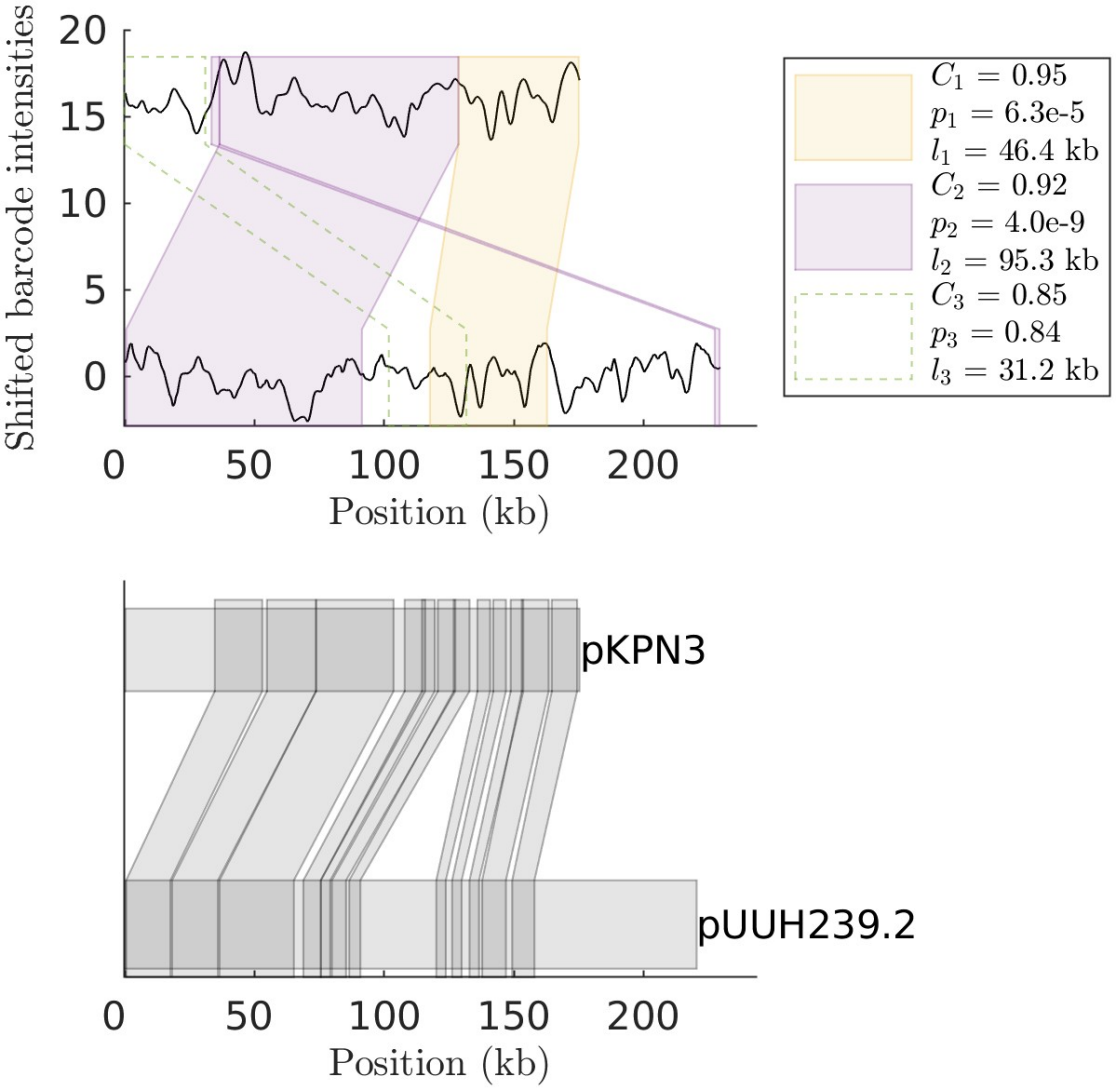
HMM output for plasmid experiment against an ancestor plasmid DNA sequence of the bacterial resistance plasmid. (Top) HMM output of an experimental consensus barcode for the pUUH239.2 plasmid compared to the theoretical DNA barcode for the ancestor (the pKPN3 plasmid). Note that we successfully identified the matching barcode-pair regions predicted by the BLAST alignment. (Bottom) BLAST output of 12 longest sub-sequence pairs with matching similarity of at least 90%.

The second example deals with the fact that plasmids are very dynamic with respect to their genomic arrangements and that plasmids found in clinical samples often are chimeras of already sequenced plasmids. Fig 6 (Bottom) shows a plasmid, referred to as pUUH239.2, that was isolated at an outbreak at Uppsala University hospital that we have previously studied with ODM [13]. The pUUH239.2 plasmid is a very good example of the dynamics of resistance plasmids [11]. The majority of the plasmid backbone is highly similar to the pKPN3 plasmid (NC_009649) while there are three regions of different origin, a region with homology to Ralstonia chromosomal DNA, a region with homologi to *E. coli* chromosomal DNA and a 41 kb resistance cassette with homology to *E coli* plasmid pEK499 (EU935739) [11]. Fig 6 (Top) shows an experimental consensus barcode of pUUH239.2 matched against the theoretical barcode for pKPN3 using our HMM pipeline. We note that our pipeline identifies regions of high similarity between the two. Comparing the sequences at a base-pair level, we visualize the true alignment table of the 12 longest matching pairs (Fig 6 (Bottom)) obtained using BLAST (Nucleotide-Nucleotide BLAST 2.6.0+). We note that the HMM pipline finds most of the matching region on the theoretical barcode. However, sub-barcode pairs seem to be extended outside the true match. This is to be expected, as the sub-barcodes can be over-extended if the intensity differences of neighbouring pixel values to the edges of the sub-barcodes are small.

Finally, we investigated computational times of the HMM pipeline. We found that for a typical case of 200kb length plasmids, the main calculation step prior to the p-value calculation takes only around 4 seconds on a standard laptop. In S10 Fig in S1 Text we show further computational times for the length range 100 to 500 kbps.

## Discussion and outlook

The goal of this study was to develop a method to directly detect SVs in barcodes from densely-labelled ODM without having access to DNA sequencing information. To that end, we introduced a Hidden Markov Model (HMM) based approach and showed that it is sufficient to use only two different types of states, gap and match states. We determined the natural values of HMM hyper-parameters using random SV barcodes, thus foregoing the need to run expensive parameter fitting procedures. We demonstrated that it is possible, using densely-labelled competitive binding DNA barcodes as scaffolds, to locally align DNA barcoding experiments in the presence of SVs. However, our procedure is applicable to any other densely-labelled ODM techniques, such as DNA melt-mapping and dense enzymatic labelling [2, 3].

We also applied a matrix profile method from time series analysis, for determining the length re-scaling factors of experimental barcodes (step 1. in our method, see Sec. 3.7 in S1 Text and S3 Fig in S1 Text) and for calculating the significance of discovered sub-barcode pairs (step 5. in our method). This shows the potential for other time series methods to be applied in the analysis of optical mapping data, thus bridging the gap between time series analysis and ODM. In particular, previous methods have shown how to find variable length motifs on a single time series [25]. Similarly, statistical significance for discords has been recently analyzed [26].

In some experimental samples, we could not estimate the initial length re-scaling factor (See Sec 3.3 and S4 Fig in S1 Text). One of the possible reasons for this was that the initial length re-scaling factor was chosen too small, as the barcodes needed to be re-scaled well beyond that factor. This shows that while our method can be successful at detecting initial length re-scaling constant, it is still a good practice to use a reference molecule of known length in the experimental assay in order to correctly estimate the nanometer-to-basepair conversion factor.

In our probabilistic post-processing approach we used a p-value threshold (step 5. in our method, see Sec. 3.5 in S1 Text) which was set to 1% here as in our previous study [20]. In applications where a different error rate is preferable, one can simply tune the p-value threshold accordingly.

A fundamental limitation in the ODM is the width of the optical point spread function (of the order 1 kb). This resolution limit sets a sharp lower bound for the lengths of sub-barcodes that we are able to detect using the present method. However, in the future combining competitive binding with sparsely-labelled ODM could potentially increase efficiency of the method.

We hope that the methodology developed herein, together with our publicly available software, will open up for routine use of densely-labelled ODM in application where detection of SVs in DNA are of importance.

## Supporting information

S1 Text Supplementary methods. Contains definitions and mathematical details of the HMM model, a description of our method for choosing the HMM parameters and of our post-processing procedure.
(PDF)
