# Supplementary material for: Detection of structural variations in densely-labelled optical DNA barcodes: A hidden Markov model approach
Source: PLoS One. 2021 Nov 5;16(11):e0259670. doi: 10.1371/journal.pone.0259670 (PMC8570516; doi:10.1371/journal.pone.0259670)
Supplement: S1 Text — Contains definitions and mathematical details of the HMM model, a description of our method for choosing the HMM parameters and of our post-processing procedure. (PDF) [file pone.0259670.s001.pdf]

Supplementary Methods: Detection of structural variations in densely-labelled optical DNA barcodes: a hidden Markov model approach

Albertas Dvirnas*[1], Callum Stewart[1,2], Vilhelm Müller[3], Santosh Kumar Bikkarolla[3,4], Karolin Frykholm[3], Linus Sandegren[5], Erik Kristiansson[6], Fredrik Westerlund[3], and Tobias Ambjörnsson[1]

[1] *Department of Astronomy and Theoretical Physics, Lund University, Lund, Sweden*
[2] *Current Address: Department of Biostatistics and Health Informatics, Institute of Psychiatry, Psychology and Neuroscience, King's College London, London, United Kingdom*
[3] *Division of Chemical Biology, Department of Biology and Biological Engineering, Chalmers University of Technology, Gothenburg, Sweden*
[4] *Current Address: School of Engineering, Engineering Research Institute, Newtownabbey, United Kingdom*
[5] *Department of Medical Biochemistry and Microbiology, Uppsala University, Uppsala, Sweden*
[6] *Department of Mathematical Sciences, Chalmers University of Technology and the University of Gothenburg, Gothenburg, Sweden*

Here we provide supplementary material including definitions and further explanations of the methods in the main text.

Table 1: Experiments and their names in the main text. Query and reference are described in the analysis pipeline.

Figure	Query	Reference	Source
Fig 5 (Top)	$P6K0$	$P6K25$	Fig.3 from [9]
Fig 5 (Middle)	$P1K0$	$P8K0$	Fig.3 from [9]
Fig 5 (Bottom)	$P6K0$ (215 kb)	$P6K22$	Fig.2 from [9]
Fig 6 (Top)	$DA15001 - pUUH\_150603$	pkPN3	
Fig S7 (Top)	P1K0	P3K0	Fig.3 from [9]
Fig S7 (Middle)	P1K0	P5K0	Fig.3 from [9]
Fig S7 (Bottom)	P11K0	P11K22	Fig.3 from [9]
Fig S8 (Top)	NC_016966_inversion_2	NC_016966.1	
Fig S8 (Middle)	$DA15001 - pUUH\_150603$	NC_016966_inversion_2	

*albertas.dvirnas@thep.lu.se (AD)

Table 2: Table of parameters related to the length re-scaling factors. Parameter $L_{\text{maxRescale}}$ determines by how much the query barcode can be re-scaled in length when searching for the estimated length re-scaling factor. L_{step} the grid step between lengths of re-scaled barcodes. L_{STOMP} is the sub-barcode length parameter used when estimating the length re-scaling factor determines. L_{HMM} is the final length re-scaling factor around the estimated length re-scaling factor.

Parameter	Name	Value
Maximum rescaling factor	$L_{\text{maxRescale}}$	10%
Step for scaling factors	L_{step}	2 pixels
sub-barcode length	L_{STOMP}	100
HMM scaling factors	L_{HMM}	2%

Table 3: Input parameters for rcOSVDP problem

Variable	Purpose
T^A	Query barcode
T^B	Reference barcode
p_{MM}	Probability of a jump from match to match state
p_{GG}	Probability of a jump from gap to gap state
c	Minimum number of consecutive match states before jumping to a gap state
cg	Minimum number of consecutive gap states before jumping to a gap state

Table 4: Match table from the HMM alignment

Start query	Stop query	Start ref	Stop ref	Orientation
135	550	1	416	1
1	83	417	499	1

Table 5: Hyperparameters used in the HMM model

Parameter name	Value	Purpose
p_{MM}	0.51	Probability of jumping from match state to match state
p_{GG}	0.31	Probability of jumping from gap state to a gap state
l	44 pixels	Minimum number of consecutive match states
l_G	0 pixels	Minimum number of consecutive gap states

Table 6: Parameters used in the post-processing of the HMM output

Parameter name	Value	Purpose
g	5	Gap length threshold
		sub-barcodes which are separated by gaps of length $\leq g$ are merged
p_{thresh}	0.01	p-value threshold

1 Definitions

An optical DNA map is a sequence-specific finite array of numbers, called intensity profile or a barcode. To make the notation consistent, we provide formal definitions of a barcode and related concepts of sub-barcodes, matching sub-barcode pairs, and the structural variation (SV) detection problem.

Definition 1. *A barcode $T \in \mathbb{R}^n$ is an array of real valued numbers $t_i \in \mathbb{R}^n$, i.e. $T = [t_1, \ldots, t_n]$, where n is the length of T.*

The barcode value t_i at position i represents photon count (including camera read out effects [1]) collected at a pixel i along the DNA molecule (i.e. positions in a nanochannel) in an optical DNA mapping (ODM) experiment.

For the detection of SVs, we are interested in how the barcode values change locally, i.e. the local properties of a barcode. Therefore, we consider arrays of real numbers of shorter length than that of a barcode, called *sub-barcodes* (in time-series analysis they are called sub-sequences, but not to confuse the terminology with DNA sequence analysis, we adopt the notation of sub-barcodes throughout):

Definition 2. *A sub-barcode $T_{i,m} \in \mathbb{R}^m$ of a barcode T with $i + m - 1 \leq n$ is a subset of consecutive values from T of length m starting from position i. Formally, $T_{i,m} = [t_i, t_{i+1}, \ldots, t_{i+m-1}]$.*

If a barcode is T is considered to be circular, then the sub-barcodes are allowed to loop around, and we define a circular sub-barcode through

Definition 3. *A circular sub-barcode $T_{i,m} \in \mathbb{R}^m$ of a barcode T with $m \leq n$ is a subset of consecutive values from T of length m starting from position i. Formally, $T_{i,m} = [t_i, t_{(i \bmod n)+1}, \ldots, t_{(i+m-2 \bmod n)+1}]$, where* mod *denotes a module operation.*

We're also interested in the reversed sub-barcode, since it might happen that the directions of two barcodes do not match:

Definition 4. *A reverse sub-barcode $\hat{T}_{i,m} \in \mathbb{R}^m$ of a sub-barcode $\hat{T}_{i,m} \in \mathbb{R}^m$ with $m \leq n$ is a subset of consecutive values from T of length m starting from position $i+m-1$ and ending at position i. Formally, $\hat{T}_{i,m} = [t_{i+m-1}, \ldots, t_i]$.*

To compare two barcodes to each other, we introduce a similarity measure *dist*:

Definition 5. *As the similarity measure $d_{i,j}$ between sub-barcodes $A_{i,m}$ and $B_{j,m}$ we define*

$$d_{i,j} = \max(pcc(A_{i,m}, B_{j,m}), pcc(\hat{A}_{i,m}, B_{j,m})),$$

where pcc is the z-normalized Pearson cross correlation coefficient defined in Algorithm 1 and $\hat{A}_{i,m}$ is the reverse sub-barcode of $A_{i,m}$.

```
function: pcc
input   : A,B
output  : dist
seq1 ← zscore(A, 1);
seq2 ← zscore(B, 1);
L ← length(A);
dist ← (1/L) seq1 · seq2';
```

Algorithm 1: Function *pcc*, which is used to compute Pearson Cross correlation function pcc between two barcodes, A and B, of the same length. *zscore* is a function which subtracts the mean and divides by standard deviation. Prime refers to a vector transpose. If we want to calculate the similarity measures *dist* by sliding barcode A along barcode B, we instead use a fast Fourier transform (FFT) based approach.

Definition 6. *A distance profile D_i of the barcode A compared to the barcode B is a vector of dist scores between the sub-barcode $A_{i,m}$ and each sub-barcode in the barcode B. Formally, in case of linear barcodes, $D_i = [d_{i,1}, \ldots, d_{i,n-m+1}]$, where $d_{i,j}$ is the similarity measure between $A_{i,m}$ and $B_{j,m}$.*

Using the definition of the distance profile, we define a matrix profile (which is a vector that calculates a profile over a matrix) for barcodes A and B:

Definition 7. *A matrix profile P between barcodes A and B is a vector of dist scores between each sub-barcodes $A_{i,m}$ and its nearest neighbour (closest match) in the barcode B. Formally, $P = [min(D_1), \ldots min(D_{n-m+1})]$, where D_i, $1 \leq i \leq n-m+1$ is the distance profile D_i of sub-barcode $A_{i,m}$ compared to the barcode B.*

A *matching sub-barcode pair* (in time-series literature, it is called pair motif) between two barcodes A and B (assumed to be of the same length n for simplicity) is defined according to:

Definition 8. *A matching sub-barcode pair of length m is the most similar pair of sub-barcodes from barcodes A and B. Formally, this is a pair of sub-barcodes $A_{a,m}$ and $B_{b,m}$, such that $dist(A_{a,m}, B_{b,m}) \geq dist(A_{i,m}, B_{j,m})$ $\forall i, j \in [1, 2, \ldots, n-m+1]$.*

Example 1. *Consider barcodes $T^1 = [1, 3, 4, 5, 6, 7]$ and $T^2 = [2, 3, 4, 5, 6, 8, 3]$. Then the matching sub-barcode pair of length $m = 4$ is a pair $T^1_{2,4} = [3, 4, 5, 6]$ and $T^2_{2,4} = [3, 4, 5, 6]$.*

We also defined a merged sub-barcode pair, in the case when two sub-barcode pairs $A_{x_1,m}$, $B_{y_1,m}$ and $A_{x_2,n}$, $B_{y_2,n}$ are merged given $x_1 \leq x_2$ and $y_1 \leq y_2$ (as in Sec. 3.1):

Definition 9. *A merged matching sub-barcode pair $A_{x_1,m}$, $B_{y_1,m}$ and $A_{x_2,n}$, $B_{y_2,n}$ is a subset of values of barcodes A and B: $[A_{x_1}, \ldots, A_{x_1+min(u_1,u_2)}, A_{x_2}, \ldots, A_{x_2+n-1}]$, and $[B_{y_1}, \ldots, B_{y_1+min(u_1,u_2)}, B_{y_2}, \ldots, B_{y_2+n-1}]$, given $u_1 = x_2 - (x_1 + m - 1)$, $u_2 = y_2 - (y_1 + m - 1)$*

We seek to find a set of sub-barcode pairs between two barcodes A and B that maps (possibly overlapping) sub-barcodes of A to non-overlapping sub-barcodes of B of constrained length such that each pair of sub-barcodes is a matching sub-barcode pair. We call this a structural variation detection problem (SVDP). The solution to this problem is a set of sub-barcodes a and b, such that they form a matching sub-barcode pair between barcodes A and B, and any two sub-barcodes from B have no common points (are disjoint).

Definition 10. *(SVDP) Find a non-empty set $S(T^A, T^B) = \{(T^A_{i,m}, T^B_{j,m}), T^A_{i,m} \subset T^A, T^B_{j,m} \subset T^B\}$, such that $(T^A_{i,m}, T^B_{j,m})$ is a matching sub-barcode pair, and $\forall T^1_{j,m}, T^2_{j,m} \in T^B$, $T^1_{j,m} \cap T^2_{j,m} = \emptyset$.*

This problem could have many possible solutions, so we define a maximization problem (alternatively a minimization problem, if instead we use Euclidean distance, i.e. $ED(a, b) = \sqrt{2n(1 - dist(a, b))}$.

Definition 11. *(OSVDP) Find a solution to SVDP which maximizes*

$$\arg\max \frac{1}{\sum |T^A_{i,m}|} \sum_{S(T^A, T^B)} dist(T^A_{i,m}, T^B_{j,m}) \cdot m$$

Here we divide the sum of lengths of matching sub-barcode pairs in the set #motifs(T^A, T^B) to weight the cases when the number of sub-barcode pairs is different. arg max means that we are looking for the argument for which the maximum value is reached.

We are not interested in small matching sub-barcode pairs, in particular smaller than length constraint k, and we therefore define a constrained optimal structural variation detection problem (cOSVDP). This constraint makes sure the number of elements $\#a$ is not less than k.

Definition 12. *(cOSVDP) Find a solution to SVDP which maximizes,*

$$arg \max \frac{1}{\sum |T^A_{i,m}|} \sum_{S(T^A,T^B) \ | \ m \geq k} dist(T^A_{i,m}, T^B_{j,m}) \cdot m$$

Finally, the experimental barcodes might have slightly different length re-scaling factors (i.e., different basepair to pixel conversion factors, up to 10%), therefore we look for a set of matching sub-barcode pairs for length re-scaled versions of barcode A,

Definition 13. *(rcOSVDP) Re-scaled constrained structural variation detection problem. In this case we maximize over the rescaled versions of A, i.e.* $A_1, \ldots, A_k$*,*

$$arg \max_{A_1,\ldots,A_k} \frac{1}{\sum |T^A_{i,m}|} \sum_{S(T^A,T^B) \ | \ m \geq k} dist(T^A_{i,m}, T^B_{j,m}) \cdot m$$

To find an approximate solution the the rcOSVDP problem, we define a Hidden Markov Model profile for the two barcodes T^A and T^B:

Definition 14. *A HMM profile for barcode* T^A *against barcode* T^B *is defined as a 4-tuple* (N, A, B, π)*, where*

- *The number of hidden states* $N = 2 \cdot q + 2$*, corresponding to* q *(the length of barcode* T^A*) forward states* $M_1, \ldots, M_q$*,* q *backward states* $\hat{M}_1, \ldots, \hat{M}_q$*, non-emitting insert state* $\hat{G}$*, and an emitting insert state* G*;*
- *A matrix of transition probabilities between the hidden states* $A = \{A(i,j), i = 1, 2, \ldots, N, j = 1, 2, \ldots, N\}$
- *Emission probabilities* $B = \{B(k,j), k = 1, \ldots, |T^B|, j = 1 \ldots, N-2, N\}$*, that describe the probabilities that* k*th pixel of* T^B *is associated to hidden state* j*.*
- *Initial state probability vector,* $\pi_i = \frac{1}{N}, i \in 1, 2, \ldots N$*.*

Example 2. *Example of the graph topology given* $q = 4$*, and* $d = 4$*.*

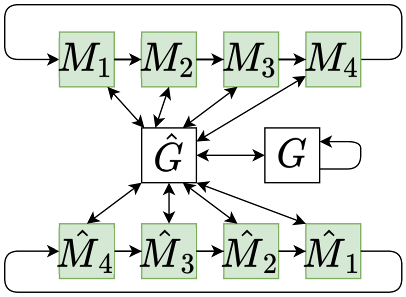

2 Methods pipeline

In this section, we go through the details of all the steps described in Materials and Methods in the main text. See also Fig 2 in the main text.

2.1 Noisified barcode generation

To estimate the efficiency of the method, we generate noisified barcodes which are similar to the experimental barcodes (Fig S1). We generate noisified barcodes of length L by adding realistically-looking noise controlled by parameter *noiseLevel* to random barcodes using Algorithm 3. This algorithm also uses Algorithm 2 to generate arrays of random normally distributed numbers and Algorithm 1 to calculate the *dist* score.

function: rand_bar
input : L
output : randBar

r $\leftarrow (r_1, \dots, r_L)$, where $r_t \sim \mathcal{N}(0,1)$;
$randSeq := [r_1, \dots, r_L] - \min(r_1, \dots r_L)$;

Algorithm 2: Function, rand_bar, for generating arrays of random numbers, randBar. $\mathcal{N}(0,1)$ is the standard normal probability density function and L is the length of the random barcode.

function: generate_noisified_barcode
input : L, psf, noiseLevel
output : $B_{\text{noisified}}$

$B_{\text{normal}} \leftarrow rand\_bar(L)$;
$N_{\text{normal}} \leftarrow rand\_bar(L)$;
$B \leftarrow imgaussfilt(B_{\text{normal}}, \text{psf}, '\text{circular}')$;
$N \leftarrow imgaussfilt(N_{\text{normal}}, \text{psf}, '\text{circular}')$;
$\alpha \leftarrow fzero(dist(B, (1-x) \cdot B + x \cdot N) + noiseLevel - 1, 0.5)$;
$B_{\text{noisified}} \leftarrow (1-x) \cdot B + N \cdot x$;

Algorithm 3: Function generate_noisified_barcode for generating noisified barcodes. This function uses Algorithm 2 to compute random barcodes. imgaussfilt is a function for convolving a vector with a Gaussian. fzero is a function that finds the value of x which solves $dist(B, (1-x) \cdot B + x \cdot N) + noiseLevel - 1 = 0$. psf is the standard deviation of the optical point spread function. *noiseLevel* is the amount of noise added to the random barcode.

2.2 Noisified random SV barcode generation

To create noisified random SV barcodes, we extend the method of noisified barcode generation to generate 5 different types of structural variations: insertions, deletions, inversions, translocations, and repetitions.

The idea behind adding an SV is that after generating an array of random numbers, we add the SV) which in case of the insertion just adds an additional array of random numbers somewhere along the initial array. This information can then be stored in a true match matrix which we call *matchTable*. This is what is done in Algorithm 4.

We then need to add the optical point spread function and noise to the array of random numbers. The whole procedure is then described in Algorithm 5. An example of random SV barcodes with all 5 main SVs that we

consider is included in the Fig 1 in the main text.

function: add_sv
input : B_{normal}, L, typeSV
output : $B_{\text{synthetic}}$, matchTable

$len1 \leftarrow length(B_{\text{normal}});$
$randPosStart \leftarrow randi(len1);$
$variation \leftarrow \text{rand\_bar(L)};$
$B_{\text{synthetic}} \leftarrow [B_{\text{normal}}[1 : randPosStart], variation, B_{\text{normal}}[randPosStart + 1 : len1]];$
$matchTable = \begin{pmatrix} 1 & randPosStart & 1 & randPosStart & 1 \\ randPosStart + 1 & len1 & randPosStart + lenVar + 1 & len1 + lenVar & 1 \end{pmatrix};$

Algorithm 4: Function add_sv, which adds a SV to the random barcode, which is generated using Algorithm 2. As an example here we show only the case where typeSV is insertion. *randi* is a random integer between 1 and *len*1. As an output we get a random SV barcode and a matchTable, which gives us the positions and orientation of the matching sub-barcodes.

function: noisified_random_sv_barcode
input : L_1, L_2, psf, noiseLevel, typeSV
output : $B_{withoutSV}$, $B_{\text{noisified}}$

$B_{\text{normal}} \leftarrow rand\_bar(L_1);$
$B_{\text{synthetic}}$, matchTable $\leftarrow add\_sv(B_{\text{normal}}, L_2, typeSV)$;
$L \leftarrow length(B_{\text{synthetic}});$
$N_{\text{normal}} \leftarrow rand\_bar(L);$
$B_{withoutSV} \leftarrow imgaussfilt(B_{\text{normal}}, \text{psf}, '\text{circular}');$
$B \leftarrow imgaussfilt(B_{\text{normal}}, \text{psf}, '\text{circular}');$
$N \leftarrow imgaussfilt(N_{\text{normal}}, \text{psf}, '\text{circular}');$
$\alpha \leftarrow fzero(dist(B, (1 - x) \cdot B + x \cdot N) + noiseLevel - 1, 0.5);$
$B_{\text{noisified}} \leftarrow (1 - x) \cdot B + N \cdot x;$

Algorithm 5: Function noisified_random_sv_barcode, which generates noisified random SV barcode. It uses Algorithm 2 for generating random barcode and Algorithm 4 to add a structural variation. L_1 is the length of query barcode, L_2 is the length of structural variation, *typeSV* is the type of the structural variation. *noiseLevel* and *psf* are described in Algorithm 3.

2.3 Length re-scaling

Barcodes from densely-labelled DNA experiments have different values for the number of base-pairs per pixel, which requires the length of one of the barcodes to be re-scaled to match the scaling of the other barcode. We perform the re-scaling using linear interpolation.

Consider a barcode $Q = [q_1, \ldots, q_k]$ of length k which we want to rescale to have length $\hat{k}$. We define $\hat{k}$ points X_i equally spaced between 1 and $k + 1$,

$$X_i = 1 + (i - 1) \cdot \frac{k}{\hat{k}}, i = 1, 2, \ldots, \hat{k} \tag{1}$$

Then we rescale barcode Q by calculating linear interpolation of points

$$[q_1, \ldots, q_k, q_1] \tag{2}$$

on a new grid defined by X_i to get a length re-scaled barcode $\hat{Q}$ (Fig S2)

$$\hat{q}_i = (q_{\lceil X_i \rceil} - q_{\lfloor X_i \rfloor})(X_i - \lfloor X_i \rfloor) + q_{\lfloor X_i \rfloor} \tag{3}$$

The algorithm associated with method described above is found in Algorithm 6 and an example of a length re-scaled barcode is shown in figure S2

function: length_rescale
input : B, L_1,L_2
output : B_{rescaled}

$B_c \leftarrow [B,\ B(1)]$;
$grid \leftarrow 1 : \lfloor L_2 + 1 \rfloor$;
$Xp2 \leftarrow 1 + (grid - 1) * L_1/L_2$;
$y = interp1(B_c, Xp2(1 : end - 1))$;

Algorithm 6: Function length_rescale, which length re-scales barcode B from length L_1 to length L_2. Here $interp1$ is matlab's linear interpolation function described in Eq.3.

2.4 Matrix profile

To compute the matrix profile between two barcodes A and B, which was defined in Sec. 1, we use a modification of the STOMP algorithm, first described in [8]. This algorithm computes a matrix profile of barcode A along B in time complexity $O(n^2)$. Our modification includes the reverse sub-barcodes: for each position along the matrix profile, we take the maximum of two matrix profiles: A versus B and A with reversed sub-barcodes versus B. This is needed since in practice barcodes might not have the same orientation.

2.5 P-value generation

In this section we describe how we calculate p-value for determined best sub-barcodes. This is used both for length re-scaling factor estimation and for final p-values for detected sub-barcodes.

We generate 1000 random barcode pairs of the same lengths as the query and reference barcode. We then run the modified STOMP algorithm (see Sec. 2.4) for sub-barcode lengths chosen to be the same as the lengths of detected sub-barcodes. This gives a maximum $dist$ for each of the 1000 random barcodes. We then fit extreme value distribution following the procedure from [6] on the vector of maximum $dist$ scores. As output, we get the extreme value distribution parameters $par1$, $par2$, see Algorithm 7. We then find the $dist$ value where $pvalue = 0.01$. These are then used to calculate p-value ($pvalue$) for the sub-barcode pair with a $dist$ score using the equation:

$$pvalue = 1 - \rho_{par1,par2}(dist) \tag{4}$$

where the cumulative distribution function is calculated as in [6]:

$$\rho_{par1,par2}(dist) = \left(\frac{1}{2}\left(1 + (-1)^{sgn(dist)} I_{dist^2}(\frac{1}{2}, \frac{par1}{2} - 1)\right)\right)^{par2} \tag{5}$$

Where $I_a(x, y)$ is the regularized incomplete beta function.

All matching sub-barcode pairs that do not pass the threshold pthresh = 0.01 are deemed to be too dissimilar and are discarded.

```
function: generate_evd_parameters
input   : len1, len2, numRnd=1000, w, psf
output  : par1, par2
for i ← 1 to numRnd do
    bar1, bar2 ← generate_noisified_barcode(len1, len2, psf, 0, 0);
    mp ← mp_profile(bar1, bar2, w);
    ccMax(i) ← max(mp);
end
par1, par2 ← compute_evd_params(ccMax) ;
```

Algorithm 7: Function generate_evd_parameters. $len1$ is length of query barcode, $len2$ is length of data barcode, $numRnd$ is the number of random barcodes used (generated using the function generate_noisified_barcode), w is the length of the sub-barcode, and psf is the standard deviation of the Gaussian used to mimic experimental point spread function, as before.

2.6 Estimating the length re-scaling factor

In the case where the initial length re-scaling constant is not known, we compare re-scaled query barcodes against reference barcodes using a matrix profile algorithm called STOMP (as described in Sec. 2.4) with a sub-barcode

length L_{STOMP} and maximum length re-scaling factor $L_{\text{maxRescale}}$. Here we choose $L_{\text{STOMP}} = 100$ pixels and $L_{\text{maxRescale}} = 10\%$ (lengths of the barcodes is re-scaled between 90% and 110%). We get a *dist* score for each length re-scaling factor (throughout the study we use Pearson Correlation Coefficient, which is defined in the Supplementary Methods). The comparison with the highest *dist* score gives the length re-scaling constant estimate L_{rescale}.

In this section we explain our choice of $L_{\text{STOMP}} = 100$ pixels (see Sec. 2.4) as a choice for sub-barcode length when calculating the estimate of the length re-scaling factor between query barcode and reference barcode. We first generate random query and noisified reference barcodes with noise $\alpha = 0.1$, and re-scale the reference barcodes by random numbers $1/s_i$ sampled from from $\mathcal{N}(1, 0.1)$. Most of the re-scaled factors calculate this way fall within the "allowed" length range determined by $L_{\text{maxRescale}}$ (See Table 2). We then compare sub-barcodes of length re-scaled query barcodes (re-scaled up to $L_{\text{maxRescale}}$) to the reference barcode using matrix profile (Section 2.4). The length re-scaling factors for which the maximum of the matrix profile is highest gives us the estimated length re-scaling factors $\hat{s}_i$, which we compare to the true length factors s_i by calculating the sum squared difference, i.e. $\sum_{i=1}^{N}(s_i - \hat{s}_i)^2$, where $N = 10$ in the experiment we run. Visual inspection of the result (See Fig S3) suggests that $L_{\text{STOMP}} = 100$ is sufficient to have a low sum squared difference between true scaling factor and detected scaling factor. To check if the detected length re-scaling factor is significant, we run the p_{thresh} calculation for sub-barcode of length L_{STOMP}, query of length $l_q \cdot \hat{s}$ and reference of length l_d. If p-value is small, we continue with considering re-scaled versions of lengths in the interval $\lfloor 0.98, 1.02 \rfloor$ (so we allow a few percent variation from the estimated length re-scaling factor). From these, we select the SV results for the length re-scaling factor which had the maximum $dist_i \cdot l_i$, where $dist_i$ is the similarity measure between longest sub-barcode pairs (of length l_i) for the length re-scaling factor i.

2.7 HMM model with k-minimum-consecutive-states constraint

We want to use the HMM to find the optimal path for the reference barcode T^B through the hidden states N in Definition 14. The optimal path describes the relation between the pixels of the query T^A and the reference T^B. A sequence of gap states G would mean that the corresponding pixel values on T^B are not likely to be similar to any pixel values on T^A, while a sequence of match states indicates a possible match. The optimal path is found by a constrained version of the Viterbi method [5] that we describe below. The algorithm (the function *compute_hmm_constrained* in the Algorithm 8) has 6 input parameters, which are summarized in Table 3. The algorithm can be divided into 3 distinct procedures - initialization (line 1 of the algorithm), which uses Algorithm 9, updating the match and gap states (loop on lines 2-12, which uses Algorithm 10 and, Algorithm 11), and finally lines 7-11 for the gap state, and a traceback (line 13, Algorithm 12).

function: compute_hmm_constrained
input : $T^A, T^B, p_{MM}, p_{GG}, c, cg$
output : score, path

1 $\delta, \psi, \xi, pM, L, S, N \leftarrow setup\_data(T^A, T^B, p_{MM}, p_{GG})$;
2 **for** $i \leftarrow 2$ **to** L **do**
3     $\xi_U, \delta, \psi, \xi \leftarrow update\_first\_element(T^A, T^B, \delta, \psi, \xi, p_M, c, cg, S, N, i)$;
4   **for** $st \leftarrow 2$ **to** S **do**
5         $\xi_U, \delta, \psi, \xi \leftarrow update\_nth\_element(T^A, T^B, \delta, \psi, \xi, p_M, c, cg, S, N, i, st)$;
6   **end**
7     $\delta(i, N) = \delta(i, N-1) + pM_{2,2} + s(0, T_i^B)$;
8     $\delta(i, N-1) = nan$;
9     $\psi(i, N) = N - 1$;
10     $\xi_U(N) = \xi_U(N-1) + 1$;
11     $\xi = \xi_U$;
12 **end**
13 $score, path \leftarrow traceback\_viterbi(\delta, \psi, L)$;

Algorithm 8: compute_hmm_constrained, main procedure of the constrained HMM algorithm. It uses Algorithms 9, 10, 11, 12 as subfunctions.

The initialization step (Algorithm 9) sets up initial values for the three arrays δ, ψ, ξ that we keep in memory through the algorithm, and the parameters pM (log of probabilities), L (length of reference), S (length of query), N (number of hidden states). $\delta(j, i)$ stores the probability that the jth pixel of D is matched one of the hidden states i. $\psi(j, i)$ stores the previous hidden state from which we move to the current hidden state i for pixel j. ξ keeps track of how many consecutive states were visited. In the first step all states are only visited once, so $\xi_1(i)$. In all calculations the probabilities are converted to log scores, which allows us to have sums in the algorithms instead of products. As scores we use the negative of the Euclidean distance, i.e. $s(x, y) = -(x-y)^2$ as we do all the calculations in the log-likelihood space.

In the next steps (lines 2-12) of Algorithm 8), we iterate through the pixels of the T^B barcode. We begin by updating the scores for the non-emitting gap state $G_{\text{non-emitting}}$ and the first match states (Algorithm 10). We first find which states were visited consecutively more than c times (line 2). It is permitted to move to the gap state $G_{\text{non-emitting}}$ for these states. We then compute $\delta(i, N-1)$, which corresponds to the probability of being in the gap state $N-1$ at step i (line 3). Lines 4-9 just update ϕ and ξ values for this state. Next, we compute the probabilities for being in the other states. These are split into two cases. In the case when we get close to the final pixels of reference barcode T^B, the conditions of minimum number of match states c or minimum number of gap states cg have to be checked (line 10). If neither of these is satisfied, we can only move from a match state to

another match state. Note that if $cg = 0$, then the condition $\xi_U(N-1) < cg$ is never satisfied, and we could for example jump from a gap state $G_{\text{non-emitting}}$ back to a match state without passing through the emitting gap state G. Finally, on the lines 34-35, we add the pixel emission scores to the states 1 and $S+1$.

The loop on lines (lines 3-6) of Algorithm 8 updates the ξ, δ, and ψ arrays for states 2 to $N-2$. Algorithm 11 is then very similar to Algorithm 10, with the main difference being that in Algorithm 11 we do not need to compute the scores for non-emitting gap state $G_{\text{non-emitting}}$, and match states follow each other consecutively, instead of looping around the circular barcodes. On lines 7-9 of Algorithm 8 we update the gap state G.

Backtracking through the arrays δ, ξ (Algorithm 12), we find the optimal path through the barcodes T^A and T^B. The final output is then converted to a match table as in Table 4

function: setup_data
input : T^A, T^B, p_{MM}, p_{GG}
output : $\delta, \psi, \xi, pM, L, S, N$

$L \leftarrow |T^B|$ - length of reference barcode;
$S \leftarrow |T^A|$ - length of query barcode;
$N \leftarrow 2 \cdot S + 2$ - number of hidden states;
$pM \leftarrow \log \begin{pmatrix} p_{MM} & p_{MG} \\ p_{GM} & p_{GG} \end{pmatrix} = \log \begin{pmatrix} p_{MM} & 1 - p_{MM} \\ \frac{1-p_{GG}}{2q} & p_{GG} \end{pmatrix}$ - logarithms of probabilities;
$\delta \leftarrow -inf(L, N)$ - initialize Viterbi scores matrix;
$\psi \leftarrow zeros(L, N)$ - initialize previous hidden state matrix;
$\xi \leftarrow ones(1, N)$ - number of consecutive states visited;
$\delta(1,:) \leftarrow [s(T_1^B, T_1^A) \ldots s(T_1^B, T_S^A), s(T_1^B, T_S^A) \ldots s(T_1^B, T_1^A), -inf, s(T_1^B, 0)]$;
$\psi(1,:) \leftarrow [1 \ldots N]$;

Algorithm 9: Function setup_data to set up data for the main algorithm. As inputs here we have normalized (subtracted mean and divided by standard deviation) query barcode T^A and reference barcode T^B, probability of jumping between match states p_{MM}, and probability of jumping between gap states p_{GG}. The outputs are δ- Viterbi scores matrix, ϕ - matrix storing hidden states that maximize Viterbi score at each position, ξ stores the consecutive number of same type of states visited, and L, S, and N give the lengths of reference, query, and number of hidden states.

function: update_first_element
input : $T^A, T^B, \delta, \psi, \xi, pM, c, cg, S, N, i$
output : ξ_U, δ, ψ, ξ

$\xi_U \leftarrow \xi$;
$k \leftarrow find(\xi(1 \ldots 2 \cdot S) >= c)$;
$\delta(i, N-1) \leftarrow \max\limits_{N,k}(\delta(i-1, N),\ \delta(i-1, k) + pM_{1,2})$;
$\psi(i, N-1) \leftarrow arg \max\limits_{N,k}(\delta(i-1, N),\ \delta(i-1, k) + pM_{1,2})$;
if $\psi(i, N-1) = N$ **then**
  $\psi(i, N-1) \leftarrow 0$
else
  $\xi_U(N-1) \leftarrow \xi(N)$
end
if $i > L - c + 1 \mid\mid \xi_U(N-1) < cg$ **then**
  $\delta(i, [1, S+1]) \leftarrow [\delta(i-1, S), \delta(i-1, 2 \cdot S)] + pM_{1,1}$;
  $\xi_U([1, S+1]) \leftarrow [\xi_S + 1, \xi_{2S} + 1]$;
  $\psi(i, [1, S+1]) \leftarrow [S, 2S]$;
else
  if $\delta(i-1, S) + pM_{1,1} > \delta(i, N-1) + pM_{2,1}$ **then**
    $\delta(i, 1) \leftarrow \delta(i-1, S) + pM_{1,1}$;
    $\psi(i, 1) \leftarrow S$;
    $\xi_U(1) \leftarrow \xi(S) + 1$
  else
    $\delta(i, 1) \leftarrow \delta(i, N-1) + pM_{2,1}$;
    $\psi(i, 1) \leftarrow N - 1$;
    $\xi_U(1) \leftarrow 1$
  end
  if $\delta(i-1, 2S) + pM_{1,1} > \delta(i, N-1) + pM_{2,1}$ **then**
    $\delta(i, S+1) \leftarrow \delta(i-1, 2S) + pM_{1,1}$;
    $\psi(i, S+1) \leftarrow 2S$;
    $\xi_U(S+1) \leftarrow \xi(2S) + 1$
  else
    $\delta(i, S+1) \leftarrow \delta(i, N-1) + pM_{2,1}$;
    $\psi(i, S+1) \leftarrow N - 1$;
    $\xi_U(S+1) \leftarrow 1$
  end
end
$\delta(i, 1) \leftarrow \delta(i, 1) + s(T_1^A, T_i^B)$;
$\delta(i, S+1) \leftarrow \delta(i, 1) + s(T_S^A, T_i^B)$;

Algorithm 10: Function, update_first_element, with input and output variables as described in Algorithm 8.

function: update_nth_element
input : $T^A, T^B, \delta, \psi, \xi, p_M, c, cg, S, N, i, st$
output : ξ_U, δ, ψ, ξ

if $i > L - c + 1 \,||\, \xi_U(N-1) < cg$ **then**
    $\delta(i, [st, st+S]) \leftarrow [\delta(i-1, st-1), \delta(i-1, S+st-1)] + pM_{1,1}$;
    $\xi_U([st, st+S]) \leftarrow [\xi(st-1)+1, \xi(st+S-1)+1]$;
    $\psi(i, [st, st+S]) \leftarrow [st-1, st+S-1]$;
else
    if $\delta(i-1, st-1) + pM_{1,1} > \delta(i, N-1) + pM_{2,1}$ **then**
        $\delta(i, st) \leftarrow \delta(i-1, st-1) + pM_{1,1}$;
        $\psi(i, 1) \leftarrow st - 1$;
        $\xi_U(1) \leftarrow \xi(st-1) + 1$
    else
        $\delta(i, st) \leftarrow \delta(i, N-1) + pM_{2,1}$;
        $\psi(i, st) \leftarrow N - 1$;
        $\xi_U(st) \leftarrow 1$
    end
    if $\delta(i-1, st+S-1) + pM_{1,1} > \delta(i, N-1) + pM_{2,1}$ **then**
        $\delta(i, st+S) \leftarrow \delta(i-1, st+S-1) + pM_{1,1}$;
        $\psi(i, st+S) \leftarrow st + S - 1$;
        $\xi_U(st+S) \leftarrow \xi(st+S-1) + 1$
    else
        $\delta(i, st+S) \leftarrow \delta(i, N-1) + pM_{2,1}$;
        $\psi(i, st+S) \leftarrow N - 1$;
        $\xi_U(st+S) \leftarrow 1$
    end
end
$\delta(i, st) \leftarrow \delta(i, st) + s(T^A_{st}, T^B_i)$;
$\delta(i, st+S) \leftarrow \delta(i, st+S) + s(T^A_{S-st+1}, T^B_i)$;

Algorithm 11: Function, update_nth_element, with input and output variables as described in Algorithm 8.

function: traceback_viterbi
input : δ, ψ, L
output : $score, path$

$score \leftarrow \max(\delta(L, 1 \ldots N))$;
$idx \leftarrow arg \max(\delta(L, 1 \ldots N))$;
$path = [L, idx]$;
for $k \leftarrow L$ **to** 2 **by**-*1* **do**
    $newV \leftarrow \psi(k, idx)$;
    $path = [path; k-1, newV]$;
    if $newV = N - 1$ **then**
        $newV = \psi(k, newV)$;
        $path = [path; k-1, newV]$;
    end
    $idx = newV$;
end

Algorithm 12: Function, traceback_viterbi, for performing trace-back through the Viterbi matrix. Input and output variables as described in Algorithm 8.

Example 3. *Consider a toy example.* $T^A = [-1, 2, 4, 5]$, $T^B = [4, 5, -1]$, $p_{MM} = 0.51$, $p_{GG} = 0.31$, $c = 0$, $cg = 0$. *Then the log probability matrix is*

$$pM = \begin{pmatrix} -0.6733 & -0.7133 \\ -2.4505 & -1.1712 \end{pmatrix}$$

and the negative distances for each pair of points, as well as for the gap states, can be depicted in a distance matrix

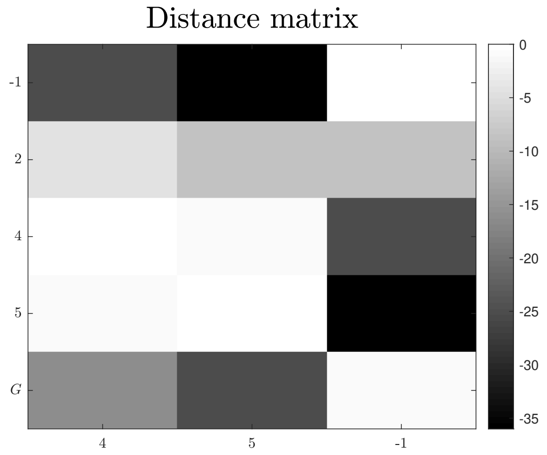

In the expected output of the HMM, the matching should start with the third element of T^A *and a first element of* T^B*. The second element of* T^A *is in this case a small insertion. There are 10 states available in this HMM. The states of this model are of three types: forward match states, labelled* M_i*, backward match states, labelled* $\hat{M}_i$*, and a gap states* $\hat{G}$*,* G*. We then run Algorithm 8 to get the* $\delta(i, j)$ *matrix:*

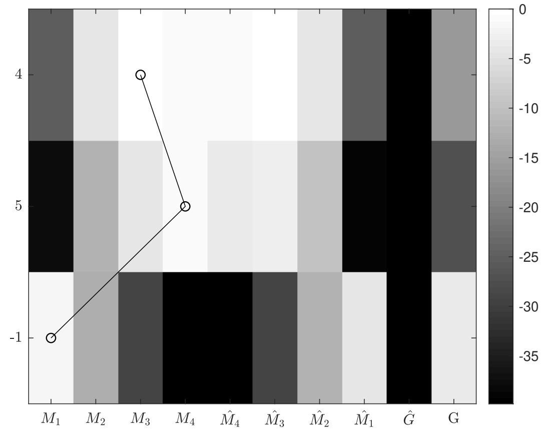

As output we get a table which matches the expected result:

Start query	Stop query	Start ref	Stop ref	Orientation
3	1	1	3	1

2.8 Robust choice of HMM parameters

In this section we describe our choice for parameters of our model. There are 4 parameters that need to be determined, i.e. jumping between states probabilities p_{MM}, p_{GG}, and the minimum number of consecutive states l, l_G. Our final choice of the constants is summarized in Table 5. Below, we describe our procedures and argumentation for this choice. Since from a match state we can jump only to a single match or a gap state, $p_{MM} + p_{MG} = 1$. From a gap state, we can either jump to another gap state or one of the $2q$ match states, therefore we have $2q \cdot p_{GM} + p_{GG} = 1$. This gives us a way to reduce the number of parameters from 4 probability parameters for jumping between states $p_{MM}, p_{MG}, p_{GM}, p_{GG}$ to 2. We choose these two independent parameters to be p_{MM} and p_{GG}.

For estimating the constants p_{MM} and p_{GG}, we run the HMM model for gridded parameter values $p_{MM} = [0.01 : 0.05 : 0.9]$, $p_{GG} = [0.01 : 0.05 : 0.9]$. As an input query barcodes we use random barcodes (of length 250 kb), and as reference barcodes we use noisified random SV barcodes (with inversion of 25 kb and noise level equal to 0.1). This gives us a true positive and true negative rates as defined in section 2.9. Averaging over the rates for 100 barcode pairs, we create heat-maps of true positive and true negative rates (See Fig S5). We use these to make a final choice for the constants p_{MM} and p_{GG}. A good choice for parameters p_{MM} and p_{GG} would be where we maximize the true positive rate, while keeping the true negative rate non-zero. Since our method is complemented by p-value threshold, most false negatives are discarded using post-processing, and therefore we make the parameter selection based on the true positive rate. As seen visually from Fig S5, the true positive rate is > 0.96 around the values $p_{MM} = 0.51$ and $p_{GG} = 0.31$, which we use as our final selection for HMM model parameters.

Finally, when selecting parameters for minimum consecutive number of states, the choice of l based on previously published result (which sets the lower bound for length of barcodes to 22 kb [6]), we choose $l = 44$ (1 pixel is approximately 500 base-pairs). For the gap states, we choose $l_G = 0$, since we want to allow jumping between non-consecutive match states without emitting a gap state. Also, we use gap merging as defined in Sec. 3.1, which makes the method insensitive to small gaps.

2.9 True positive rate and true negative rate

In this section we give an example on how true positive rate is calculated.

Example 4. *Consider a simple true alignment table*

Start ref	Stop Ref	Start Query	Stop Query	Orientation
1	25	1	25	1
26	50	50	26	2

and the corresponding match table from the HMM alignment

Start ref	Stop Ref	Start Query	Stop Query	Orientation
1	25	1	25	1
26	50	1	27	2

We find from the first row of the ground truth table that $[1, 2, \ldots, 25]$ *is matched to* $1 : 25$ *in both ground truth and HMM alignment, giving 25 true positive match pairs. Now from the second row, elements* $[26, 27, \ldots, 50]$ *from the ground truth table are matched to* $[27, 28, \ldots, 50, 1]$*. Since for each pixel we can find the nearest neighbour within one pixel (26-27),(27-28) and so on, we find that the all the pixel pairs are also true positives. Therefore the true positive rate is* $truePositiveRate = 1$*.*

For the true negative case, we use random barcodes which have no similarities, i.e. the true alignment table is empty. The ground truth matrix is also empty, and the calculation of the true negative rate reduces to counting the number of pixels of the reference barcode D that were matched somewhere (false positives, FP). True negatives are the pixels of D that were not matched anywhere. Then the true negative rate is calculated

$$trueNegativeRate = \frac{TN}{FP + TN}$$

2.10 Generating barcodes from real data

Here we follow the methodology in [7] to generate barcodes and experimental consensus barcodes. Briefly, a number of kymographs is given as an input. The kymographs are aligned and then time averaged, and we get individual experimental barcodes as an output. The individual experimental barcodes are re-scaled to the same average length, and then consensus barcode is generated using a hierarchical-clustering-types algorithm. Finally, if available, the same procedure is repeated for kymographs of lambda-DNA molecules to determine the initial length re-scaling factor. In this case after the kymographs are time averaged, re-scaled versions of the individual barcodes are compared to the theoretical barcode of lambda-DNA molecule to detect a more accurate initial length re-scaling factor.

3 Post-processing of the HMM output

In this section we discuss the details of the post-processing steps of the method. In Table 6 are the parameters used for post-processing, i.e. the gap length g and the p-value threshold p_{thresh}.

3.1 Table merging

SVs on DNA barcodes sometimes appear fragmented due to sources of error in choosing the length re-scaling factor and noise in the experimental consensus barcodes. We correct for these by disallowing $2 \cdot psf$ overlaps (approximately 5 pixels) and insertions between the sub-barcodes. We merge such sub-barcodes by removing the gaps in case of gaps between the pixels, and removing overlap pixels in case there are overlaps.

Example 5. *At a first step, we check which rows of the alignment result table should be merged. Consider the following data table:*

180	226	1	47	1
230	273	49	92	1
275	6	93	136	1
14	57	143	186	1
60	106	187	233	1
111	164	235	288	1

We find that rows 1-2-3 have to be merged together, as well as rows 4-5-6. Rows 3-4 we do not merge, because the gap is more than 5 pixels.

The merged table then only contains two rows:

180	6	1	136	1
14	164	143	288	1

Supplementary Figures

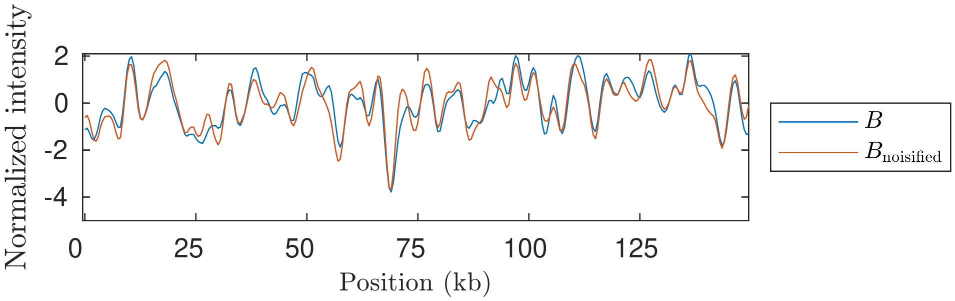

Figure S1: **Example of a noisified barcode** (Top) We generate a noisified barcode B of 300 pixels (150 kb) length, together with a noisified barcode $B_{\text{noisified}}$, such that $noiseLevel = 0.1$, i.e. the Pearson Correlation coefficient between B and $B_{\text{noisified}}$ is $C_{\text{dist}} = 1 - noiseLevel$.

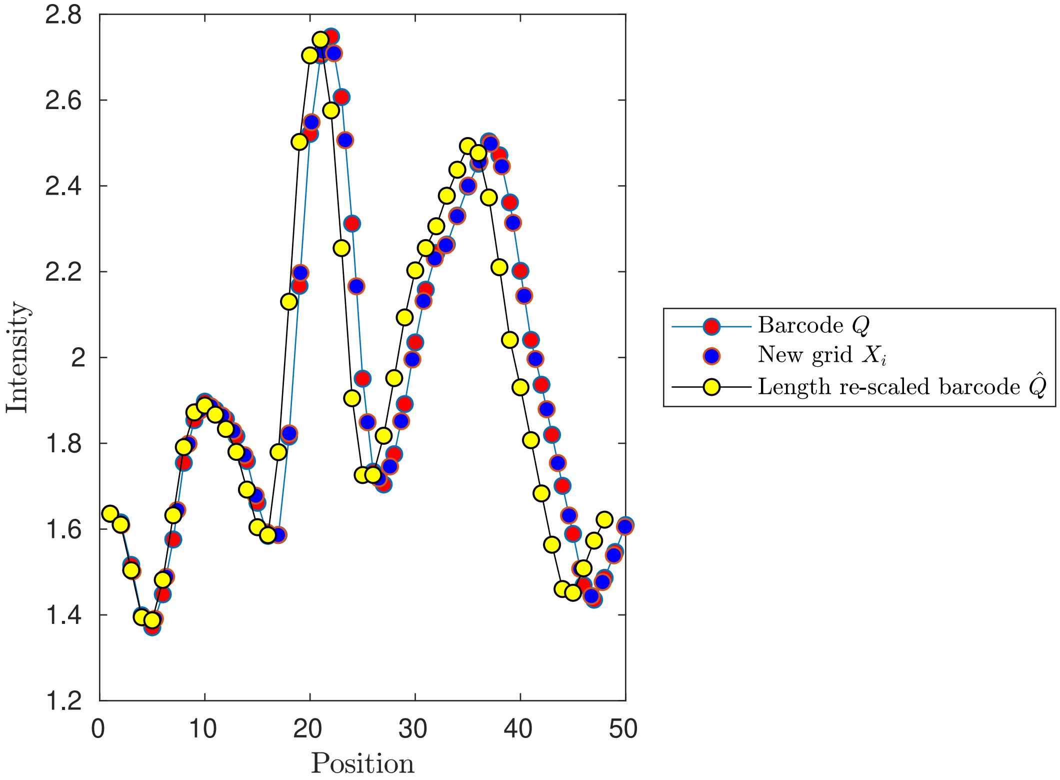

Figure S2: **Example of length re-scaling** A random barcode Q is length rescaled by 0.95 lenght re-scaling factor by linear interpolation to a grid X_i to get a length re-scaled barcode $\hat{Q}$. The mathematical details are found in Algorithm 6 .

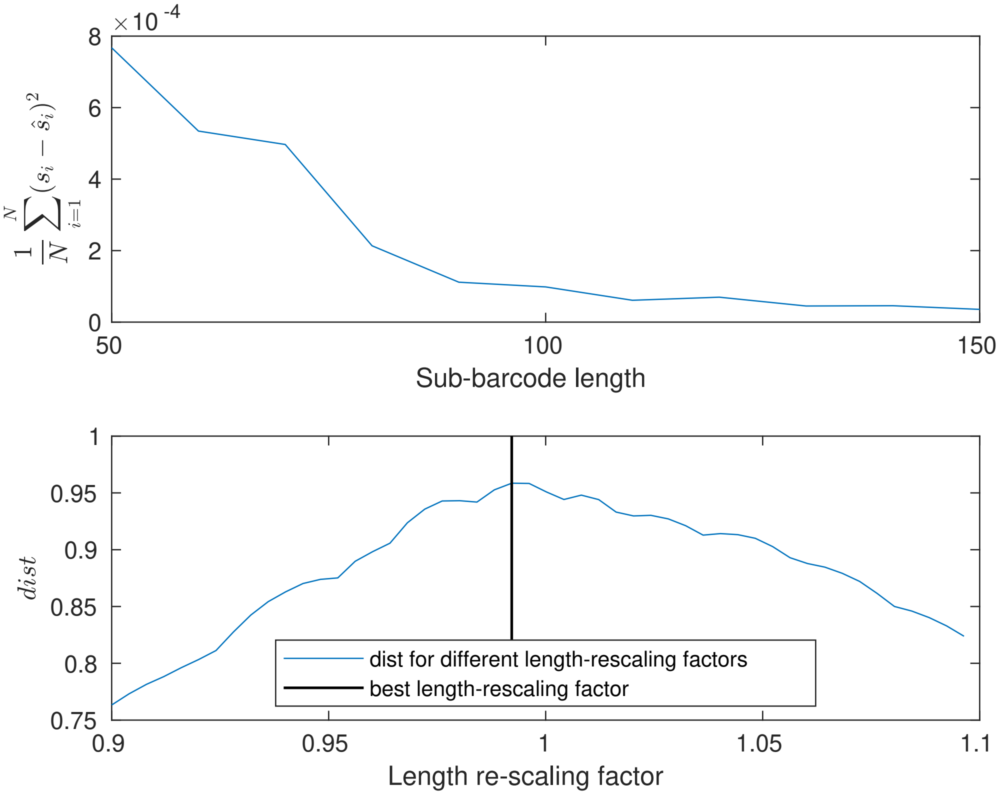

Figure S3: **Sum square difference between true and approximated length re-scaling factors** (Top) We generate pairs of 10 barcodes of 500 pixels length with noise $\alpha = 0.1$ for sub-barcode lengths of 50 to 150 pixels and at length re-scaling factors with standard deviation of 10% stretching. We compare the sum square difference between the original length re-scaling factor and the detected length re-scaling factor. Sub-barcodes of 100 pixels length or longer perform best for estimating the length re-scaling factor. (Bottom) We estimate the length re-scaling factor for a single pair of query and reference barcodes.

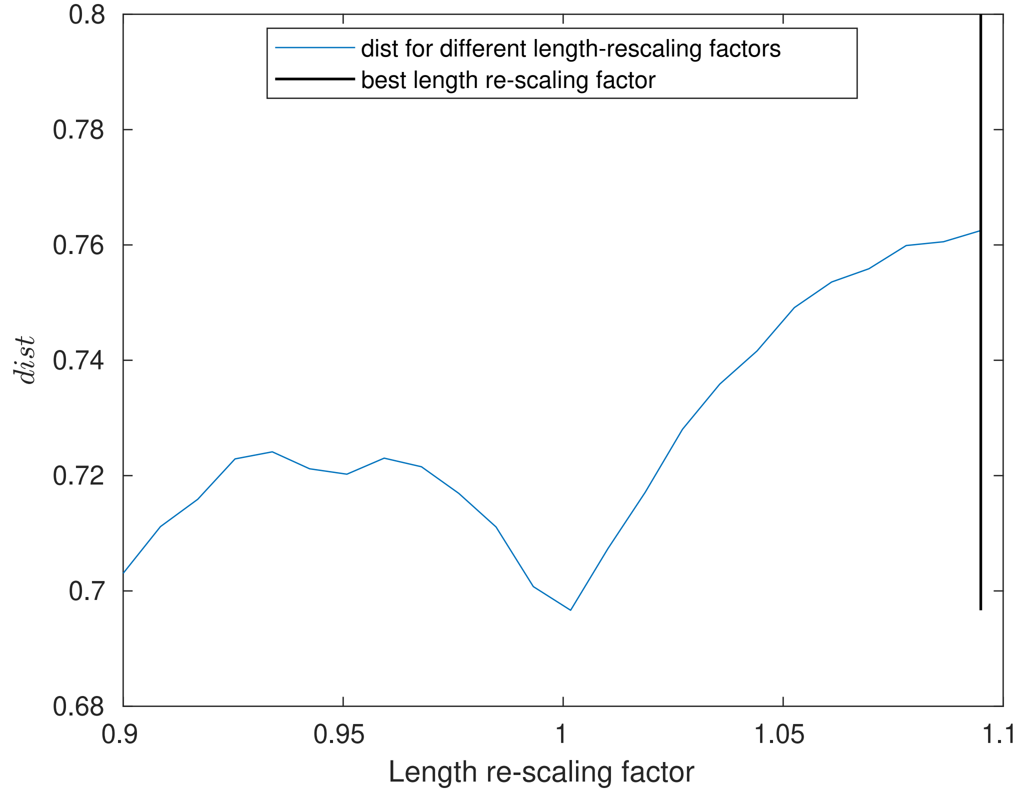

Figure S4: **Effectiveness of p_{thresh} for discarding inaccurately calculated length re-scaling factors.** Estimating the length re-scaling factor between two experimental consensus barcodes (See Table 1) gives a length re-scaling factor which is at the edge of the allowed length re-scaling factors. The *dist* score for this length re-scaling factor then does not pass the p_{thresh}, which means that the two experimental consensus barcodes are not "similar" enough to have matching sub-barcodes.

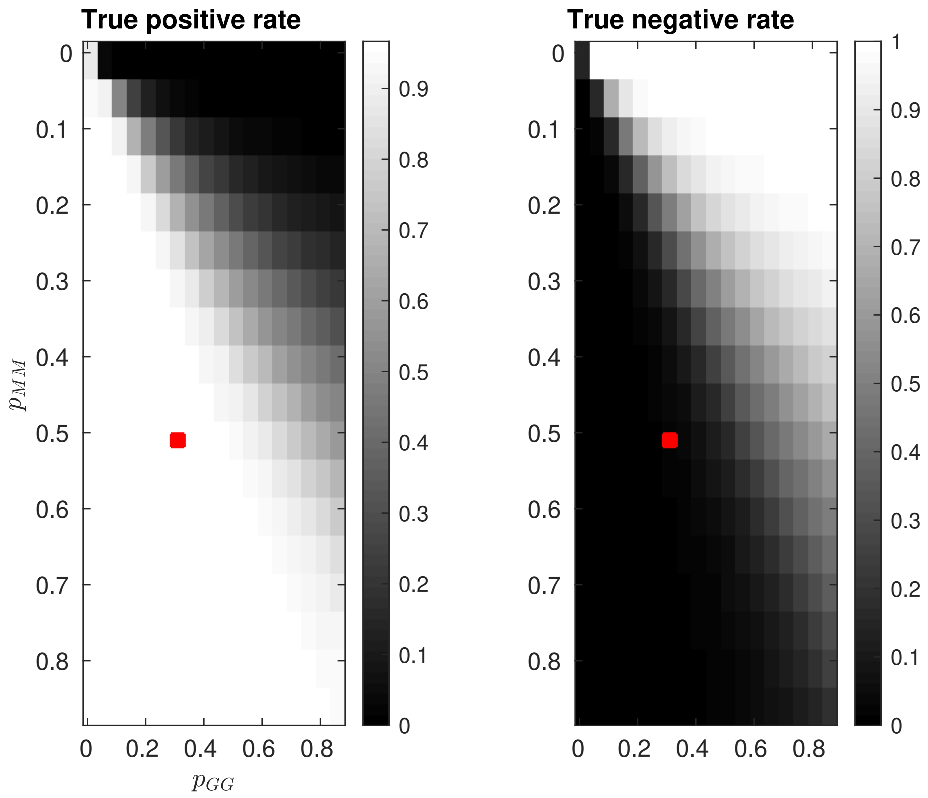

Figure S5: **Heatmap for parameter robustness analysis.** We vary the values of the HMM parameters p_{MM} and p_{GG} on a grid. For each choice of parameter, we compute the true positive rate of the HMM algorithm for 100 synthetic barcodes of length 500 pixels with a 50 pixel inversion. The red mark indicates our choice of parameters, $p_{MM} = 0.51$ and $p_{GG} = 0.31$.

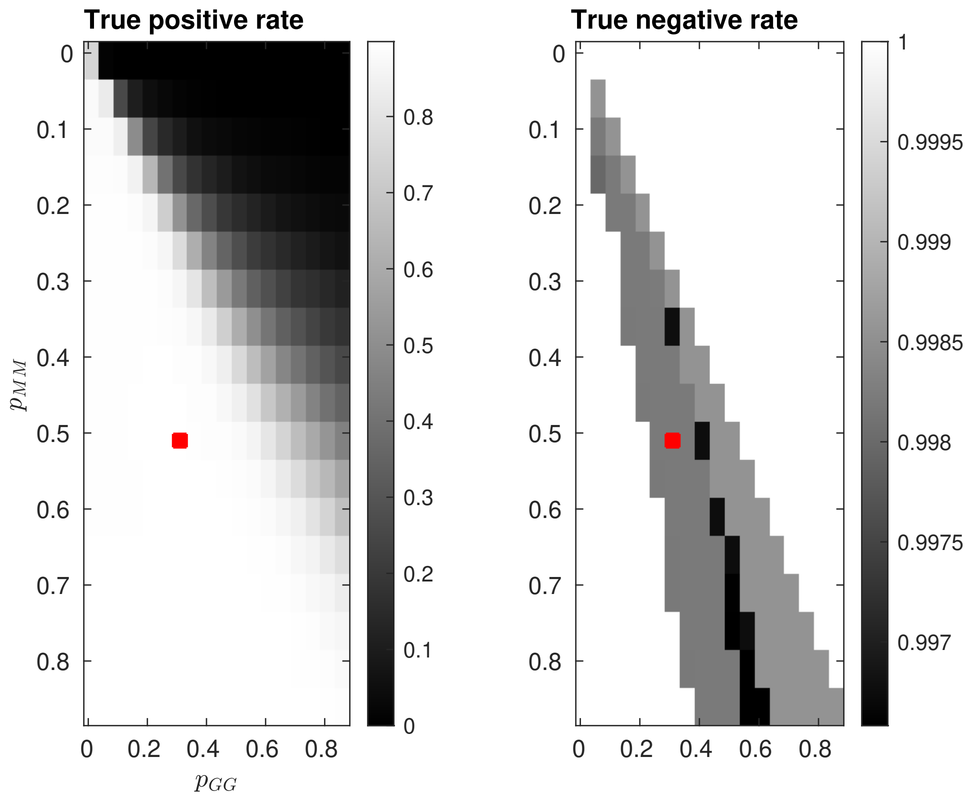

Figure S6: **Heatmap for parameter robustness analysis after p-thresh.** We vary the values of the HMM parameters p_{MM} and p_{GG} on a grid. For each choice of parameter, we compute the true positive rate of the HMM algorithm after p-thresh for 100 synthetic barcodes of length 500 pixels with a 50 pixel inversion. The red mark indicates our choice of parameters, $p_{MM} = 0.51$ and $p_{GG} = 0.31$.

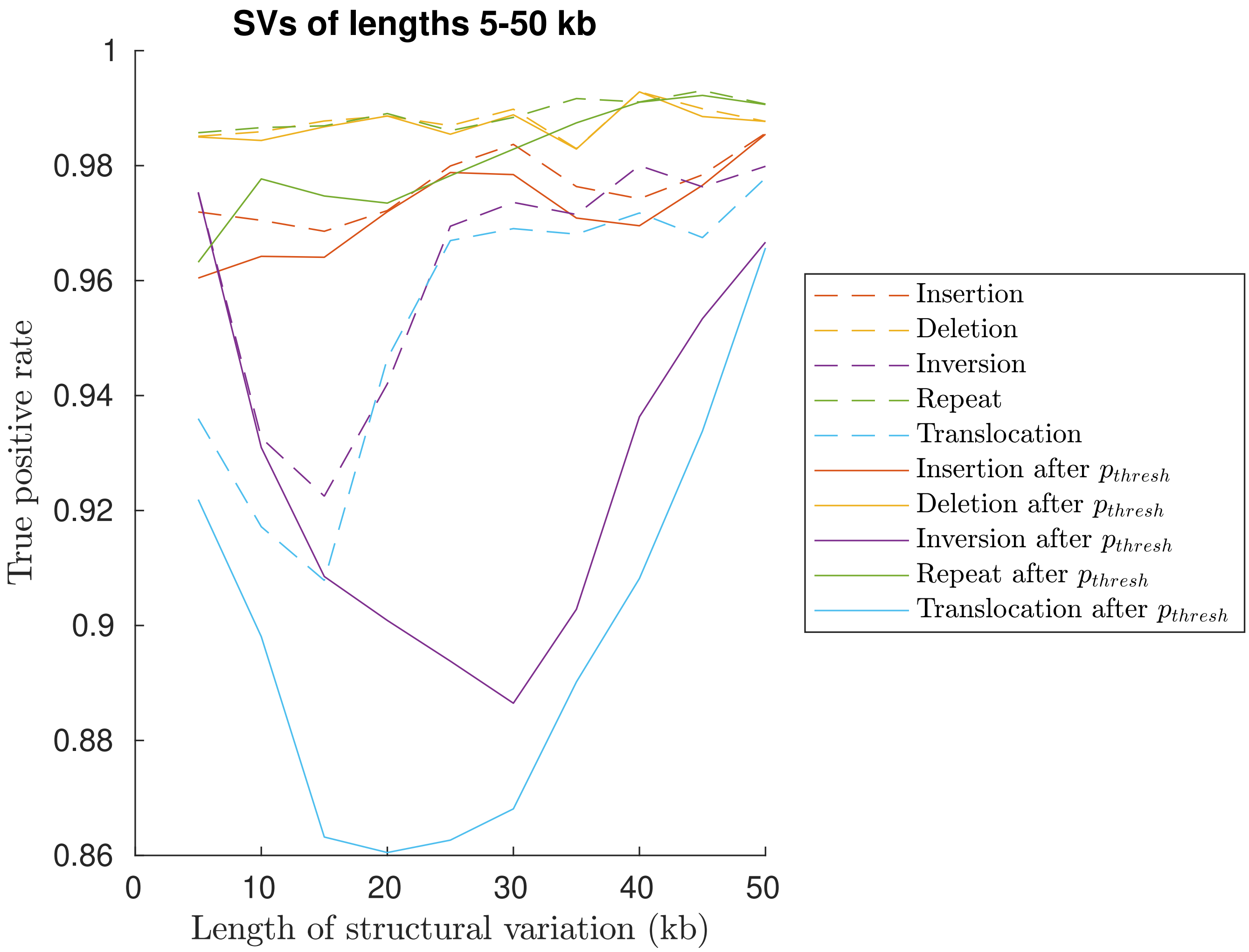

Figure S7: **Dependence of true positive rate on lengths of SVs in synthetic barcodes of different SVs.** We evaluate 5 different SVs (insertion, deletion, inversion, repeat, and translocation) with synthetic query and reference barcodes to test how true positive rate depends on the presence of SVs of different lengths. The associated figure testing the dependence in a presence on different level of noise is found in main text, Figure 5.

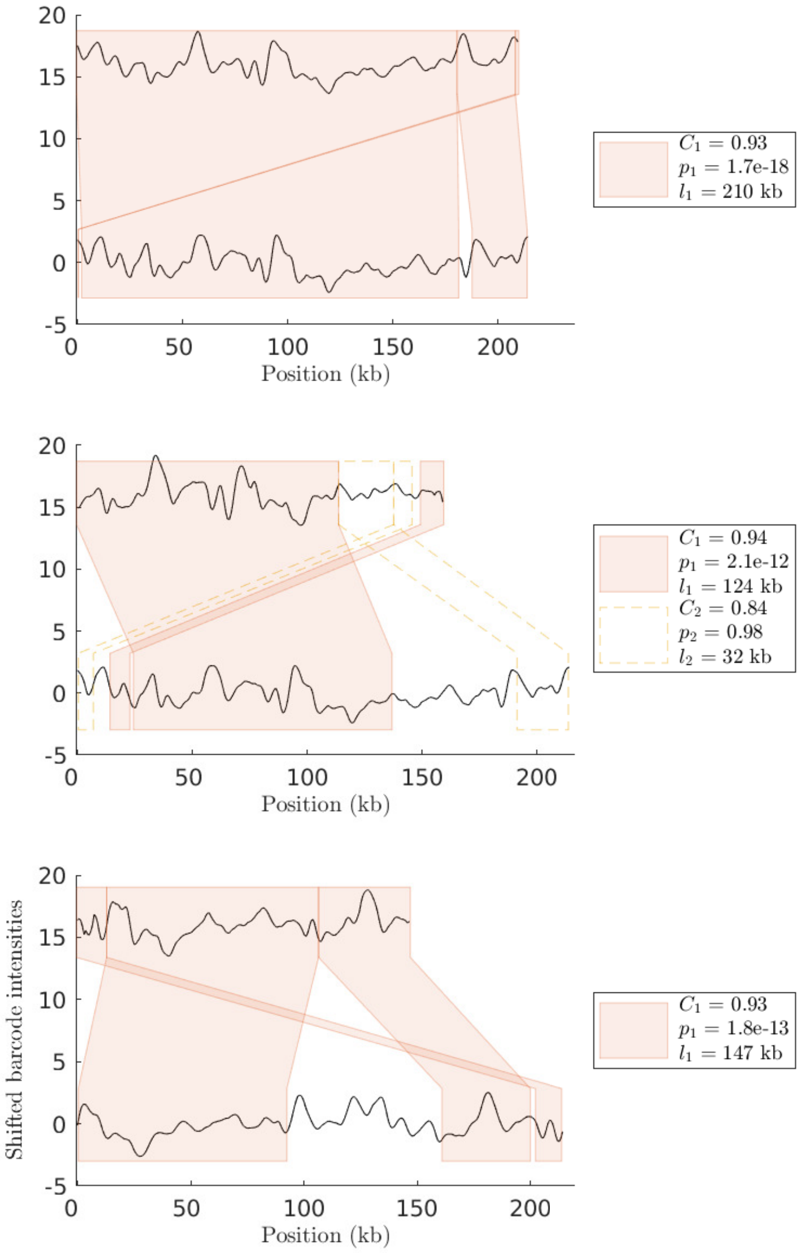

Figure S8: **HMM output for real data from a neonatal outbreak[9].** Output of the HMM method for comparison of experimental ESBL-KP 215 kb consensus barcodes. (Top) Detected sub-barcode pairs suggest that there was a roughly 5 kb deletion. (Middle) Detected sub-barcode pairs suggest that there was a 59 kb deletion, but since one of the sub-barcode pairs was not significant, this is inconclusive. (Bottom) Output of the HMM method for comparison of two experimental 215 kb consensus barcodes which shows a change that occurred within a patient over a 2 years period, suggests that there was a 68 kb deletion. Same color boxes contain significantly matching sub-barcodes. The detected sub-barcode has a *dist* score C_i, p-value p_i, and is of length l_i.

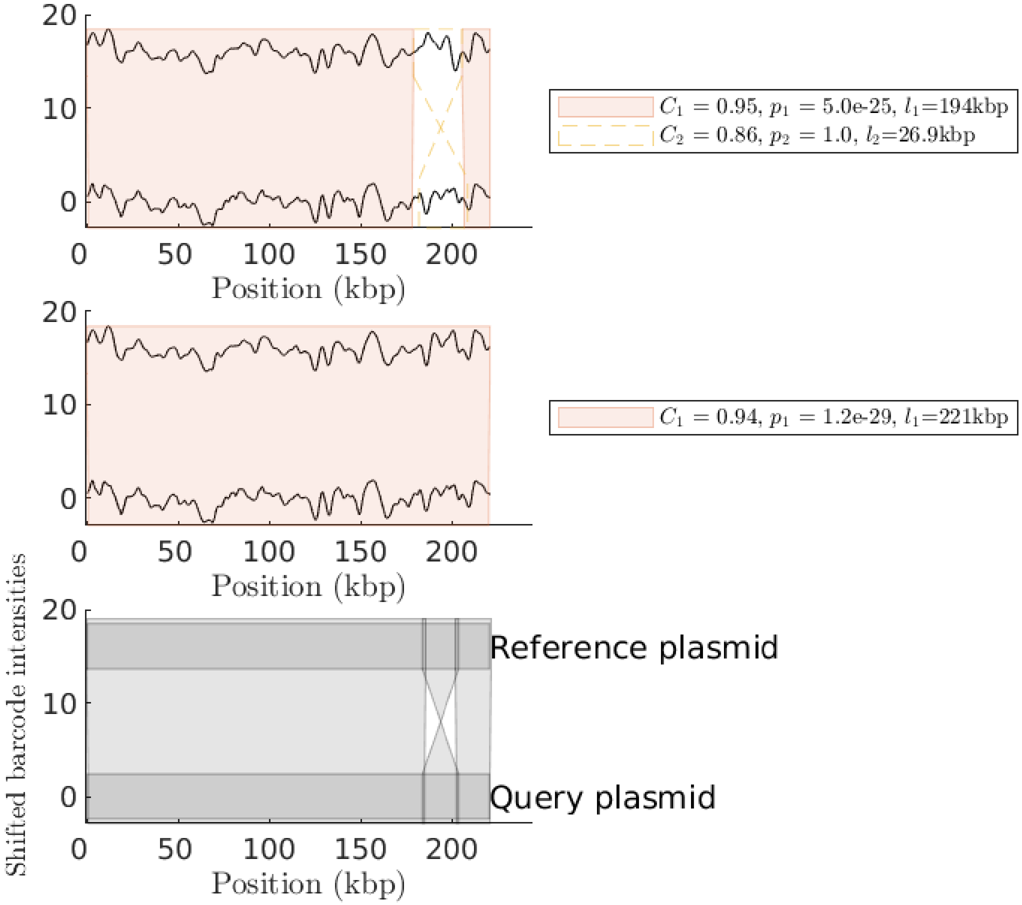

Figure S9: **HMM output for plasmid experiment against two theoretical barcodes of bacterial resistence plasmid DNA sequences.** Output of the HMM method for comparison of experimental consensus barcode to theoretical barcodes. (Top) Experimental barcode as query compared to the first theoretical barcode. (Middle) Experimental barcode as query compared to the second theoretical barcode. (Bottom) Visual comparison of two theoretical barcodes using an alignment table calculated by running BLAST on the corresponding sequences.

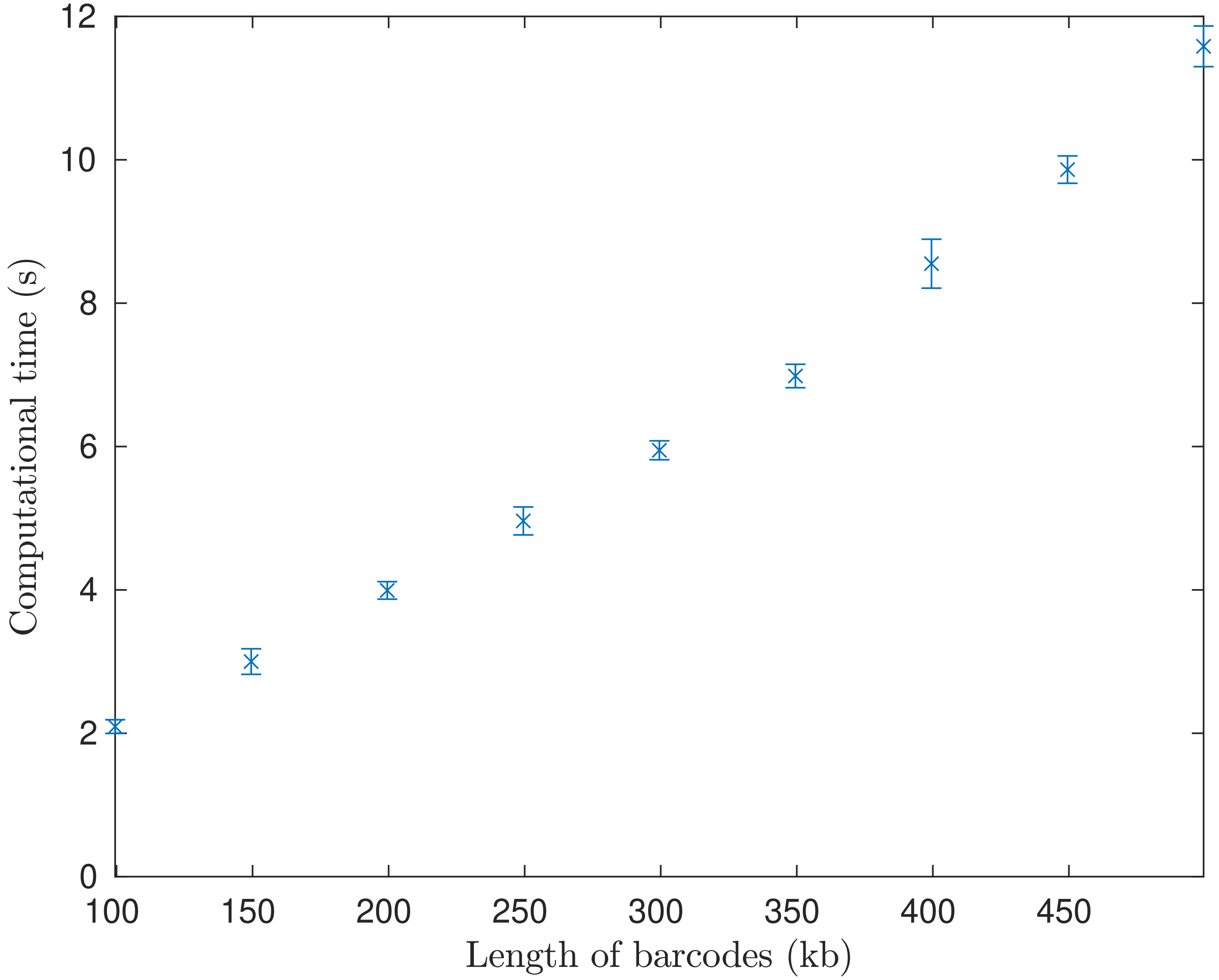

Figure S10: **Running time of the HMM on different data sets.** We performed a running time analysis of the HMM method for barcode pairs with a single inversion. The barcodes had different lengths from 100 kb to 500 kb, and for each length we ran 10 different examples to compute the mean and standard deviation of computational time. The calculation was done on a Thinkpad X1 Carbon (2nd Gen) laptop.

References

[1] Hirsch M, Wareham RJ, Martin-Fernandez ML, Hobson MP, Rolfe DJ. A stochastic model for electron multiplication charge-coupled devicesfrom theory to practice. PloS one. 2013 Jan 31;8(1):e53671.

[2] Zhu Y, Zimmerman Z, Senobari NS, Yeh CC, Funning G, Mueen A, Brisk P, Keogh E. Matrix profile ii: Exploiting a novel algorithm and gpus to break the one hundred million barrier for time series motifs and joins. In2016 IEEE 16th international conference on data mining (ICDM) 2016 Dec 12 (pp. 739-748). IEEE.

[3] Transell, M. (2012). The Use of Bioinformatics Techniques to Perform Time-barcode Trend Matching and Prediction.

[4] Stewart, C. (2017). Methods for structural variation detection and improved theory prediction for densely labeled DNA barcodes.

[5] RABINER, Lawrence R. A tutorial on hidden Markov models and selected applications in speech recognition. Proceedings of the IEEE, 1989, 77.2: 257-286.

[6] Dvirnas, Albertas, et al. Facilitated sequence assembly using densely labeled optical DNA barcodes: A combinatorial auction approach. PloS one, 2018, 13.3: e0193900.

[7] Mller V, Dvirnas A, Andersson J, Singh V, Kk S, Johansson P, Ebenstein Y, Ambjrnsson T, Westerlund F. Enzyme-free optical DNA mapping of the human genome using competitive binding. Nucleic acids research. 2019 Sep 5;47(15):e89-.

[8] Yeh CC, Zhu Y, Ulanova L, Begum N, Ding Y, Dau HA, Silva DF, Mueen A, Keogh E. Matrix profile I: all pairs similarity joins for time series: a unifying view that includes motifs, discords and shapelets. In2016 IEEE 16th international conference on data mining (ICDM) 2016 Dec 12 (pp. 1317-1322). Ieee.

[9] Bikkarolla SK, Nordberg V, Rajer F, Mller V, Kabir MH, Sriram KK, Dvirnas A, Ambjrnsson T, Giske CG, Navr L, Sandegren L. Optical DNA Mapping Combined with Cas9-Targeted Resistance Gene Identification for Rapid Tracking of Resistance Plasmids in a Neonatal Intensive Care Unit Outbreak. MBio. 2019 Aug 27;10(4):e00347-19.
